# Future effects of climate and land-use change on terrestrial vertebrate community diversity under different scenarios

**DOI:** 10.1098/rspb.2018.0792

**Published:** 2018-06-20

**Authors:** Tim Newbold

**Affiliations:** Centre for Biodiversity and Environment Research, Department of Genetics, Evolution and Environment, University College London, Gower Street, London WC1E 6BT, UK

**Keywords:** biodiversity, climate change, future, global, land-use change, scenarios

## Abstract

Land-use and climate change are among the greatest threats facing biodiversity, but understanding their combined effects has been hampered by modelling and data limitations, resulting in part from the very different scales at which land-use and climate processes operate. I combine two different modelling paradigms to predict the separate and combined (additive) effects of climate and land-use change on terrestrial vertebrate communities under four different scenarios. I predict that climate-change effects are likely to become a major pressure on biodiversity in the coming decades, probably matching or exceeding the effects of land-use change by 2070. The combined effects of both pressures are predicted to lead to an average cumulative loss of 37.9% of species from vertebrate communities under ‘business as usual’ (uncertainty ranging from 15.7% to 54.2%). Areas that are predicted to experience the effects of both pressures are concentrated in tropical grasslands and savannahs. The results have important implications for the conservation of biodiversity in future, and for the ability of biodiversity to support important ecosystem functions, upon which humans rely.

## 1. Introduction

Increasing effort and resources have been invested in conserving biodiversity globally, but most measures of biodiversity suggest continuing decline in the face of increasing human pressures [1]. Of the many pressures that humans exert on biodiversity, use of the land for agriculture and settlements is one of the most important [2]. It has been estimated that land use has caused ecological assemblages to lose on average 13.6% of species compared with globally pristine habitats [3].

Climate change is emerging as an increasingly important driver of biodiversity change, and has already had substantial effects on the structure of ecological assemblages [4,5]. Model predictions have suggested that many species will lose a substantial proportion of their distribution, or even become extinct globally as a result of predicted future climate change [6,7]. On the other hand, it has been hypothesized that the diversity of ecological assemblages could increase as a result of climate change, if the species that benefit from climate change become much more widespread and move into new habitats [8].

Most studies of the effects of climate change have focused on measures of species endangerment [6,7]. However, there is an increasing interest in how human activities are changing the diversity and structure of local ecological assemblages [3,9,10], largely because local diversity is an important determinant of the functioning of ecosystems [11–13]. There are more direct measures of ecosystem functioning than species richness, such as functional diversity [14], but species richness is widely used because it is relatively simple to estimate. Syntheses of empirical information have suggested that ecosystem function is substantially impaired where more than 20% of species are lost [11]. Previous studies have estimated that levels of biodiversity loss have exceeded this level across 28% of the terrestrial surface as a result of land-use and human-population change alone [3] (as much as 58% if the effects of roads are also included [15]). Climate change is likely to add to this figure.

How future climate change and land-use change will combine globally to alter the diversity of ecological assemblages remains poorly understood [16]. Models of climate-change effects (species distribution models) are based on point-occurrence data for species [6,7] that are spread very patchily across the globe [17], and are often resolved too coarsely to permit an accurate matching with land-use data [18]. As a result, global studies of the impacts of climate change on species have often relied on expert-drawn extent-of-occurrence distribution maps for those species [19–21]. Inclusion of land use in these projections has been rare [20,21], and has relied on expert classifications of the habitat preferences of each species, which are available for a minority of species globally and classify habitat only as suitable or unsuitable. Other studies modelled the effects of climate change, but simply overlaid predictions of land-use change without considering the sensitivity of biodiversity to different land-use types [22]. By contrast, the quantification and prediction of land-use impacts has been based on syntheses of local-scale studies [3]. Collated data from local-scale studies are not comparable along sufficiently wide climatic gradients to understand climate-change impacts. An alternative approach to understand combined land-use and climate effects is to unite the strengths of global synthetic models of the effects of land use on local biodiversity [3] and distribution models estimating the effects of climate [7].

In this study, I make the first global predictions of the separate and combined (additive) effects of future climate and land-use change on local vertebrate biodiversity. I use an ensemble of species distribution models [6] to assess climate impacts, and a recently published statistical model of land-use impacts [3].

## 2. Material and methods

### (a) Models of climate impacts

To assess climate impacts on species, I use species distribution models, which relate estimates of the presence or absence of species in different locations to environmental variables [23]. Distribution models have the advantage of being easily applied to many species, but omit important processes that might influence responses of species to climate change [24]. More sophisticated mechanistic models that incorporate these processes are prohibited by the lack of information for most species globally. The distribution models were based on published, expert-drawn distribution maps for amphibians, reptiles and mammals [25,26]. I excluded from these raw range map areas where species are considered vagrant, and areas where species are present only in the non-breeding season or during migration. I then further refined the range maps to exclude areas outside the known elevational limits of species, where available (see electronic supplementary material for data sources).

I used four climate variables, commonly reported to show a good association with vertebrate distributions (e.g. [27]): minimum temperature of the coldest month, total annual precipitation, growing degree days and water balance. Minimum temperature of the coldest month and total annual precipitation were taken directly from the WorldClim Version 1.4 dataset [28]. Growing degree days and water balance were calculated based on other climatic variables from WorldClim, using previously published methods [29].

I resampled all of the distribution maps and climate data onto a 10 km×10 km equal-area grid, which was used for the fitting of all distribution models. All grid cells that intersected some part of a species’ distribution polygon were considered to be occupied and were considered as presence points in the distribution models, to avoid very narrowly distributed species being discounted from the analysis.

I fitted distribution models using five methods commonly used in other studies [23,30]: Maxent, generalized linear model (GLM), random forest (RF), Bioclim and Domain. Using an ensemble of modelling approaches often leads to more accurate predictions [31]. I chose methods that allow the fitting of relatively simple functional forms, and used only four climatic variables, to avoid as much as possible overfitting the coarse-scale distribution maps used. Random forests fit the most complex functional forms, and are thus probably most prone to overfitting. However, their predictions were central among the ensemble members, and so did not strongly influence the final average results. For all modelling approaches, I discounted species that occupied fewer than 10 analysis grid cells. Upper limits on the number of occupied grid cells were also imposed for some modelling approaches (see detailed methods); data for species with more than the maximum number of records were subsampled randomly.

To evaluate the accuracy of each distribution model, using the area under the receiver operating characteristic curve (AUC) statistic, I divided the dataset into 80% for calibration and 20% for evaluation. Although AUC values do not always behave desirably for assessing distribution model accuracy [32], they are a simple, generally applicable measure of model accuracy, widely used in broad-scale applications of distribution models [20]. I considered models with an AUC value greater than 0.8 to be useful for making future projections. Models with this level of accuracy were generated for 20 932 species using Maxent (80% of vertebrate species with available range maps, 68% of all described vertebrate species [33]), 20 932 using GLM (80%, 68%), 20 938 using RF (80%, 68%), 18 184 using Bioclim (69%, 59%) and 17 876 using Domain (68%, 58%).

### (b) Models of land-use impacts

The models of land-use impacts were based on data in the PREDICTS database [34,35] (electronic supplementary material, figure S1), which contains published data on the composition of assemblages in different land uses. The vertebrate data consisted of 479 642 occurrence or abundance records for 6184 species (20% of all described terrestrial vertebrate species [33]), sampled at 7585 locations spanning all terrestrial biomes except one (flooded grasslands and savannahs).

For this study, I modelled just one measure of local assemblage biodiversity: inferred species richness (for the models of land-use response, the sum of all species recorded at each site). Although measuring biodiversity only as species richness may not capture all important facets of biodiversity change [36], species richness remains a widely used metric [3,9]. Furthermore, there is no simple monotonic relationship between the predicted climatic suitability from species distribution models and other measures of biodiversity, such as abundance [37]. Species richness was modelled as a function of three measures of human pressure at each site [3]: land use [34], land-use intensity [34] and human population density [38], using generalized linear mixed-effects models with a negative binomial distribution of errors, fitted using the glmmADMB package version 0.8.4 in R. I fitted a random intercept of study identity to control for among study variation in sampling methods, sampling effort and focal taxonomic group [3].

### (c) Climate and land-use scenarios

I applied my models to the representative concentration pathway (RCP) scenarios [39], the most recent global set of scenarios for which both climate and land-use estimates were available at the time of this study. The land-use projections are likely to be conservative because they make optimistic assumptions about future agricultural yields [39], and ignore some of the ways in which land use impacts biodiversity [16,40]; nevertheless, they remain the best available at a global scale. There are four RCP scenarios, which make widely differing assumptions about future socio-economic pathways (see electronic supplementary material, table S1). RCP 8.5 has been characterized as ‘business as usual’ [3,41] and most closely matches recent trends in greenhouse gas emissions [42].

I obtained future climate estimates from WorldClim version 1.4 [28] at a spatial resolution of 5 arc-minutes. Projections of land use were taken from the land-use harmonization project [43]. Land-use predictions are generated by a single model for each RCP scenario, precluding a consideration of model uncertainty in the predictions [44]. The land-use projections consist of estimates of the proportion of each terrestrial grid cell, at a spatial resolution of 0.5°, in each of 6 major land-use classes: primary vegetation, secondary vegetation, plantation forest, cropland, pasture and urban. To divide secondary vegetation according to stage of recovery—young, intermediate and mature—I followed the methods in ref. [3]. Estimates of land-use intensity and human population density were also obtained as in ref. [3] (see also the detailed methods).

### (d) Projections of climate-impact models

Projection of the distribution models onto future climate estimates produces estimates of the relative climatic suitability of each grid cell. I converted this raw projection to a binary prediction of presence or absence using a threshold that minimizes the difference between model sensitivity and specificity, which has been shown generally to perform well [45].

I projected species distributions for the reference time period (1961–1990) and each of the two future time periods (2041–2060 and 2061–2080), and for each of the four RCP scenarios of climate change (see above). For each of the 12 scenario–time period combinations, I made one of three assumptions about species ability to disperse in response to changing climatic suitability. For all projections (current and future) and all dispersal scenarios, I assumed that species could not move beyond occupied combinations of biome and biogeographic realm, making the somewhat unrealistic assumption that major habitats would not shift in response to climate changes within the time period simulated. The least conservative dispersal scenario assumed unlimited dispersal ability, where a species was assumed to be able to occupy all climatically suitable areas. The most conservative dispersal scenario assumed no dispersal ability, with species only able to occupy areas that were suitable in the reference time period and that remained suitable in future time periods. Finally, I simulated an intermediate dispersal scenario (termed here, the ‘realistic’ scenario) assuming that species could move at a specified rate away from suitable areas in the reference time period, using clade-specific rates of dispersal: 0.5 km year^−1^ for reptiles and amphibians, and 3 km year^−1^ for mammals and birds. These rates correspond with the ‘optimistic’ clade-specific dispersal rates assumed in a previous study of vertebrate responses to climate change [6]. Dispersal rates vary substantially within these major clades of species [46–48], but species-specific estimates are not yet available for most vertebrate species. The final distribution model projections for all dispersal scenarios were resampled (using bilinear interpolation) to 0.5° resolution to match the land-use projections.

Overall projections of species richness change under climate change were calculated for each grid cell by summing the species for which the cell was estimated to be climatically suitable, and then expressing this species richness as a percentage of the value for the same grid cell in the reference time period (1961–1990). Identifying an appropriate reference condition for climate projections is challenged by the fact that climate has changed constantly, but the use of 1961–1990 averages is standard practice [6]. Uncertainty in the climate projections was estimated as the full range of projected species richness values across the five model types in the ensemble.

### (e) Projections of land-use-impact models

The projections of land-use impacts on biodiversity assumed that the average local species richness across a given grid cell, as a percentage of the species richness estimated to occur naturally, is a simple multiplicative function of the proportional area and relative biodiversity values of the different land use and land-use intensity combinations within a grid cell [3]. The effect of human population density was estimated assuming that humans were distributed uniformly throughout a grid cell. In reality, human population density probably covaries with land use and land-use intensity, but the scenario data did not permit this level of detail in the projections. Uncertainty estimates (95% confidence intervals) for the projections of land-use impacts were derived from the uncertainty in the coefficients from the mixed-effects models of land-use responses. Species richness for the land-use projections was expressed as a percentage relative to a reference condition where all habitat is minimally disturbed primary vegetation with a human population density of zero.

### (f) Combining the land-use and climate projections

The projections of land-use and climate impacts on local species richness were combined assuming that the pressures act on species independently of each other. Global averages for the combined projections of land-use and climate impacts (as well as for the projections of the effects of the pressures individually) were calculated as the average across all terrestrial grid cells, weighted by cell area and by total ‘natural’ vertebrate species richness, i.e. in the absence of any climate or land-use impacts. Total natural species richness was estimated by overlaying the available extent-of-occurrence range maps—as described above—for all modelled species.

### (g) Modelling limitations

Both the species distribution models and land-use response models have limitations and uncertainties [49,50]. A comparison and combination of these approaches, such as I present here, is complicated by the fact that the models reflect processes operating at very different spatial scales. However, each model is currently the best available to address either climate or land-use effects, and both models make predictions about the average change in local species richness across a landscape. Therefore, while a precise comparison of the predicted effects of land use and climate is problematic, it is possible to compare the predicted magnitudes of the effects, and to identify the scenarios and locations associated with the largest effects of each pressure.

The assumption that the effects of climate and land use are independent of one another is unlikely to hold for a number of reasons. First, climate and land use might disproportionately impact a similar set of species. For example, narrow-ranged species have been shown to be disproportionately sensitive to land use [51], and are likely to be the most sensitive to climate change. Second, climate has been shown—at least at small scales—to influence how species respond to land use [52,53]. Third, land use might influence the ability of species to disperse through landscapes in response to climate change [49]. At present, there is insufficient information to account properly for these interactions, but they should be included in more refined models in future.

## 3. Results

**Figure 1.**
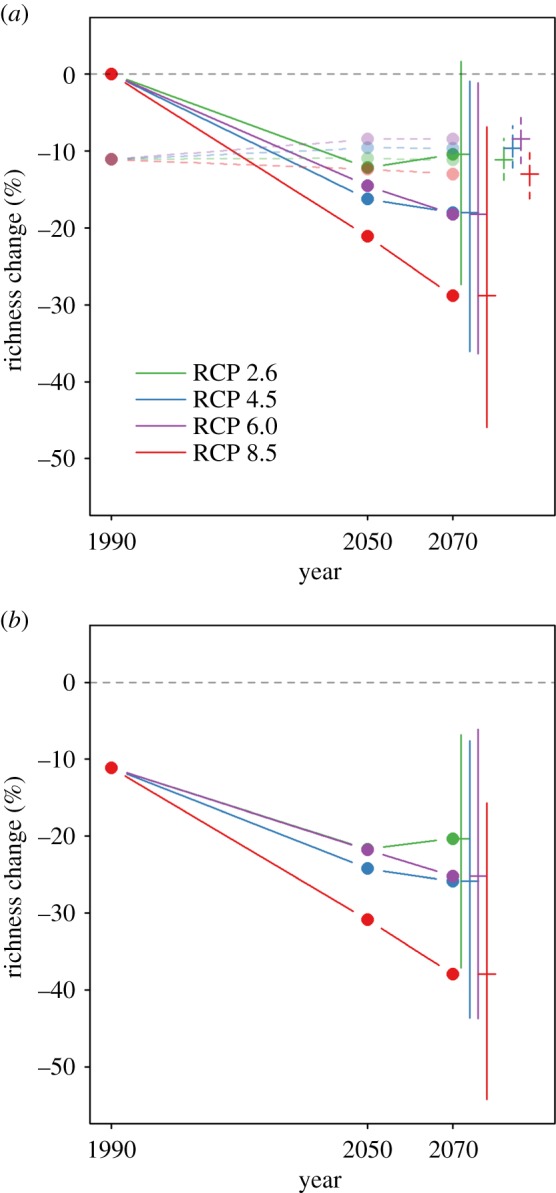
Predicted species richness of ecological communities under future climate and land-use change. All values are expressed relative to a pre-human baseline. Separate effects of climate (solid, opaque lines) and land use (dashed, translucent lines) are shown in (*a*), while combined effects of both pressures (assuming no interactions) are shown in (*b*). Error bars show estimated uncertainty in the projections for the year 2070: 95% confidence intervals for land-use impact models, range of estimates across the distribution model ensemble for the climate impact models and combined (additive) uncertainty for the combined projections. Results for both land-use and climate impacts are based on the final projections at a spatial resolution of 0.5°.

**Figure 2.**
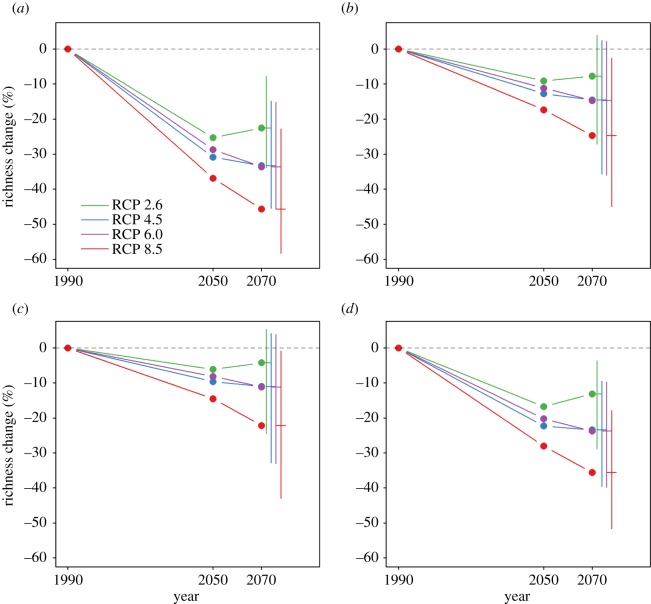
Predicted species richness of ecological communities under future climate change, for separate vertebrate clades. Predictions are shown for (*a*) amphibians, (*b*) birds, (*c*) mammals and (*d*) reptiles. Error bars show estimated uncertainty in the projections for the year 2070, expressed as the range of estimates across the distribution model ensemble.

Historical land-use changes are estimated to have caused vertebrate communities to lose 11.1% of species compared with pristine habitats (95% confidence intervals: 5.0% to 16.0%; figure 1*a*). Future effects of land use are predicted to be relatively small, ranging across scenarios from a 2.6% gain to a 1.9% further loss (95% confidence intervals: 6% gain to 5.1% loss; figure 1*a*). The RCP 2.6 scenario (electronic supplementary material, table S1), which predicts the smallest level of future climate change, led to the second most negative land-use impacts on biodiversity. RCP 8.5, which predicts the largest amount of climate change, was associated with the largest negative impact on biodiversity from land-use change (figure 2).

The results of the distribution model projections predict that climate change will become a major driver of change in the local richness of ecological communities in the coming decades. The effects of climate change are clearly predicted to match or exceed land use in its effects on vertebrate community diversity by 2070 (figure 1*a*). Even if the predictions of future land-use effects are conservative (e.g. because of optimistic assumptions about future agricultural yields [39]), the effects of climate change are predicted at least to reach levels comparable with those of historical land use. By 2070, assuming intermediate dispersal abilities, vertebrate communities are predicted to lose between 10.5% (RCP 2.6) and 28.8% (RCP 8.5) of their species locally as a result of climate change (figure 1*a*). The uncertainty in predicted climate impacts on biodiversity was large (ranging across all scenarios and all models in the ensemble from a 1.6% gain to a 45.9% loss). Most of this uncertainty depended on the distribution modelling algorithm used, and the ranking of scenarios was very similar across algorithms (electronic supplementary material, figure S2).

Different assumptions about species' ability to disperse in response to climate change had a substantial effect on predicted biodiversity change (electronic supplementary material, figure S3). Assuming no dispersal is possible, predicted average losses ranged from 35.4% to 61.0% (range across ensemble: 14.4% to 70% loss). Assuming unlimited dispersal ability, predicted losses ranged from 5.0% to 16.0% (range across ensemble: 7.5% gain to 34.3% loss; electronic supplementary material, figure S3). For all distribution model types and assumptions about dispersal ability, predicted effects of climate change were—unsurprisingly—smallest under RCP 2.6 and largest under RCP 8.5, in agreement with the results of previous studies [6].

Under the combined effects of climate and land use, vertebrate community diversity is predicted to have decreased substantially by 2070 under almost all combinations of socio-economic scenarios and assumptions about species’ dispersal ability. Assuming intermediate dispersal ability, vertebrate communities are predicted to lose, on average, between 20.3% and 37.9% of species compared with reference conditions (with combined model and scenario uncertainty, predicted losses ranged from 6.8% to 54.2%; figure 1*b*).

**Figure 3.**
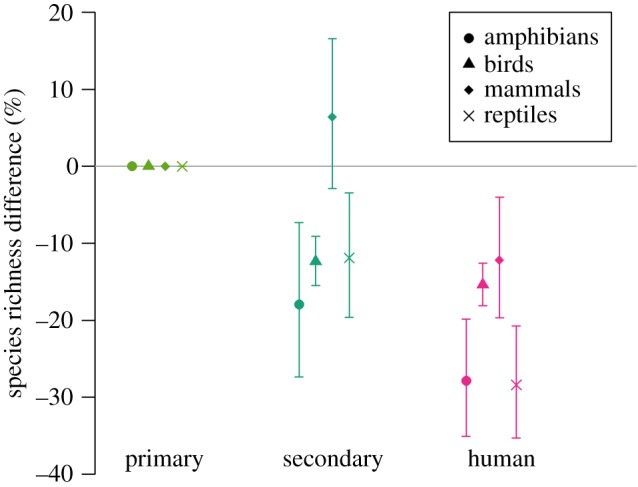
Response of each major vertebrate clade to land use. Unlike the main models of the response to land use, which combined all vertebrate classes and thus used data for many more sites (electronic supplementary material, figure S7), a much simpler classification of land use was used here, with categories of primary vegetation (pristine habitat with no record of destruction), secondary vegetation (natural habitat recovering after some recorded historical destruction) and human (plantations, croplands, pastures and areas of human settlement). Error bars show 95% confidence limits around the modelled responses. (Online version in colour.)

Amphibians and reptiles are predicted to be lost disproportionately from communities as a result of climate change compared with mammals and birds (figure 2). I caution though that the result for reptiles might be biased, given that the reptile species for which there are published range maps are only a subset of the whole clade. My results show that reptiles and amphibians are also disproportionately sensitive to human land uses (figure 3). However, there were insufficient data to project the future impacts of land-use change on each vertebrate clade separately.

**Figure 4.**
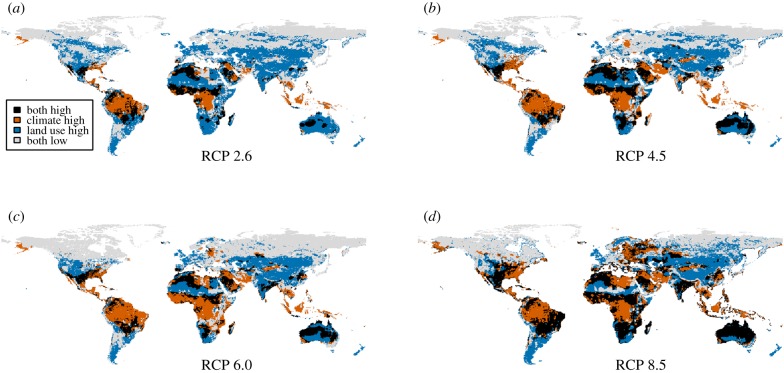
Spatial patterns of biodiversity loss from climate and land-use change by 2070. Areas with more than 10% net loss of species from climate change are shown in brown; areas with more than 10% loss of species from land-use change in blue; areas where more than 10% losses from each pressure overlap are shown in black; and areas with less than 10% losses from both pressures are shown in grey. Projections are shown for each of the socio-economic and greenhouse-gas-emission scenarios: (*a*) RCP 2.6; (*b*) RCP 4.5; (*c*) RCP 6.0 and (*d*) RCP 8.5. Results for both land-use and climate impacts are based on the final projections at a spatial resolution of 0.5°.

The predicted effects of climate change and land-use change varied spatially with different patterns. There are large parts of the world where both pressures are expected to combine, especially in tropical grasslands and savannas, and on the edge of the tropical forests (figure 4). By contrast, the centres of the tropical forests and northern boreal regions are predicted to remain less affected by land-use changes, but tropical forests are predicted to experience substantial losses of biodiversity through climate change (figure 4). Temperate regions are predicted to experience relatively small biodiversity changes from future climate change (figure 4).

**Figure 5.**
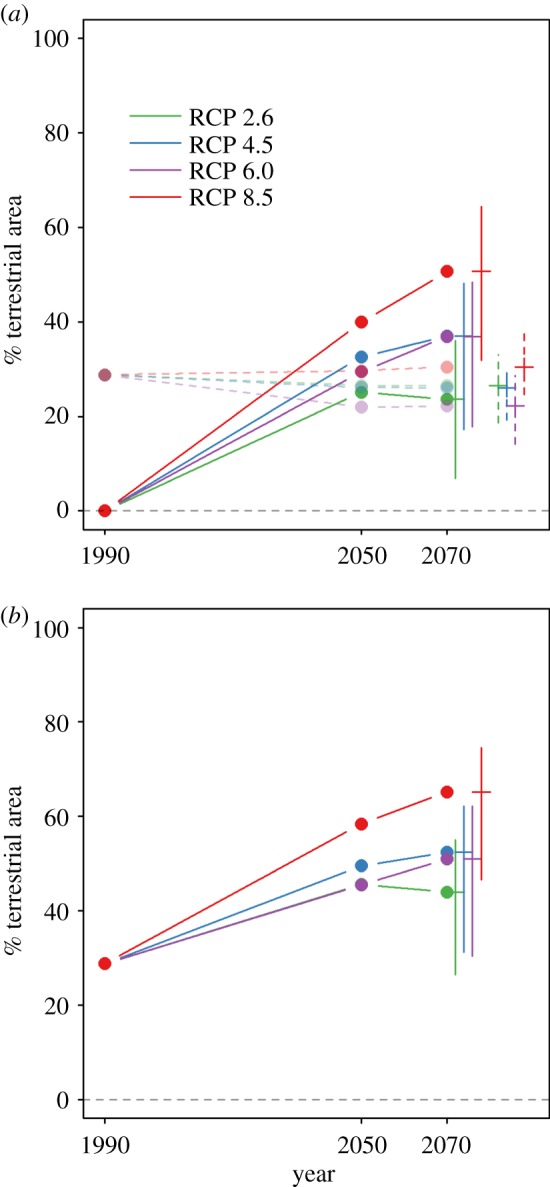
Predicted percentage of the Earth's terrestrial surface exceeding 20% species loss under future climate and land-use change. All values are expressed relative to a pre-human baseline. Separate effects of climate (solid, opaque lines) and land use (dashed, translucent lines) are shown in (*a*), while combined effects of both pressures (assuming no interactions) are shown in (*b*). Error bars show estimated uncertainty in the projections for the year 2070: 95% confidence intervals for land-use impact models, and range of estimates across the distribution model ensemble for the climate impact models. Results for both land-use and climate impacts are based on the final projections at a spatial resolution of 0.5°.

Historical land-use changes are estimated to have caused 20% loss of local species richness across 28.8% of the world's terrestrial surface (figure 5; electronic supplementary material, figure S4). This figure is predicted to increase to 30.4% or decrease to 22.2% as a result of future land-use change depending on the socio-economic scenario (95% confidence intervals: 14.1% to 38.7%) (figure 5; electronic supplementary material, figure S4). Climate change is predicted, by 2070, to have reduced local species richness by more than 20% across 23.6% to 50.7% of the terrestrial surface (range across scenarios and model ensemble: 6.9% to 64.4%). Both pressures together are predicted to reduce local species richness by more than 20% across 43.9% to 65.2% of the terrestrial surface (combined uncertainty across all scenarios: 26.4% to 74.6%) (figure 5; electronic supplementary material, figures S4 and S5).

## 4. Discussion

Overall, my results suggest that climate change will have a rapidly increasing effect on the structure of ecological communities in the coming decades, probably equalling or surpassing the effects of land use by the 2070s. My projections of climate impacts ignored any effects on vertebrate biodiversity brought about by shifts in the distribution of major biomes, and so actual effects of climate change are likely to be even greater. The predictions of future impacts of land use may be rather conservative given the optimistic assumptions the global land-use scenarios make about future agricultural yields [39,40]. Even if this is the case, climate change is predicted by the 2070s to surpass the effects of historical land use in changing the species richness of ecological communities.

Individually, the predicted effects of climate and land-use change varied among the different future scenarios considered. Unsurprisingly, climate change is predicted to have the smallest effects under a scenario with strong climate mitigation (RCP 2.6). By contrast, and as in a previous study with a greater coverage of taxonomic groups [3], RCP 2.6 was the second worst scenario for land-use impacts, largely because climate mitigation is assumed to be achieved through the rapid expansion of biofuel plantations [3]. The RCP 8.5 scenario, which assumes the largest greenhouse gas emissions (and consequently the most rapid climate change) and the greatest increase in the human population (leading to rapid expansions of human land uses), led to the most negative outcomes for biodiversity as a result of both land-use and climate change. This scenario is most closely aligned with current trajectories [3,41,42], suggesting that without changes to energy and land-use systems, future losses of local biodiversity will be large.

The combined effects of land-use and climate change could lead to the average ecological community losing more than half of its species under ‘business as usual’ (RCP 8.5), compared with non-impacted communities. Overall, the scenario with strong (mainly biofuels-based) climate mitigation still led to the least negative outcomes for biodiversity when both climate and land-use change were considered, but relatively less so than for the climate-only projections owing to the negative effect on biodiversity of biofuel expansion. A precise ranking of the different scenarios based on their predictions for climate and land-use change is problematic because of spatial-scale issues, as described above. Furthermore, the projections of land-use effects are likely to be conservative [39], in which case the benefit of the RCP 2.6 scenario, relative to the other scenarios, may be reduced or even removed. Strong, biofuels-based climate mitigation may still be better overall for biodiversity than allowing rapid climate change (although species of conservation importance might be lost in tropical regions [54], a possibility that my models do not capture). However, strong climate mitigation through other means (for example, the establishment of carbon markets that leads to the preservation of natural forest), which was not considered in the four scenarios analysed here, would likely be even more beneficial for biodiversity. My projections of the combined effects of land-use and climate change assumed no interactions between these pressures. However, there are several likely ways that land-use and climate effects will interact, including effects of land use on dispersal of species in response to climate change [55], and changes in species' responses to land use because of local climatic differences [53,55]. Understanding how such interactions might alter projections of future changes in biodiversity is a very important area of research [55].

Land-use and climate effects were predicted to vary markedly among the four major clades of vertebrates. Predicted future changes in local species richness as a result of climate change were much greater for amphibians and reptiles than for birds and mammals, similar to the findings of a previous study [6]. I also show, for the first time globally, that amphibians and reptiles respond more negatively to human land use than birds and mammals. Although there were insufficient data to make projections of land-use impacts on individual vertebrate classes, my results suggest that the combined effects of future land use and climate change will have a disproportionately strong impact on reptile and amphibian communities.

The predicted effects of changes in land use and climate also varied spatially. Tropical forests, which have seen lower rates of conversion to human land use than other areas, are predicted to experience large losses of species as a result of future climate change (figure 4). By contrast, temperate regions, which have seen some of the largest historic losses of biodiversity from land use [15], are predicted to experience relatively small biodiversity changes from future climate change (figure 4). Tropical grasslands and savannahs are predicted to experience strong losses of species as a result of both climate change and land-use change, supporting calls for more work on the conservation of these relatively under-studied ecosystems [56]. The spatial distributions of climate-change impacts presented here are very similar to those presented in two earlier studies [6,22], but contrast with a study that made projections of distribution changes of birds and found more pronounced effects at high latitudes, although using different climate scenarios [21].

Net losses of species from ecological communities in excess of 20% are thought to be associated with the degradation of key ecosystem functions and processes [11], although species richness is only a proxy for ecosystem functioning, and other measures such as functional diversity are likely to be more direct [14]. Historical land use is estimated to have caused net species richness losses exceeding this level over more than a quarter of the world's terrestrial surface, as shown previously [3]. This figure is predicted to increase or decrease slightly by the 2070s, depending on the socio-economic scenario followed, with the largest increases under the ‘business as usual’ RCP 8.5. Climate change is predicted by the 2070s to have caused a 20% net loss of species across a similar or larger proportion of the world's surface, emphasizing that climate change is likely to become an increasingly important pressure restructuring ecological communities.

Overall, my results suggest that climate change will be increasingly important in restructuring ecological communities in the coming decades. The combined effects of climate and land-use change (historical and future) are likely to cause a loss of biodiversity sufficient to have substantial negative effects on ecosystem functioning across a large proportion of the terrestrial biosphere. Efforts to identify more beneficial scenarios for biodiversity that have focused either on climate or land use [3,6] obscure the complete picture. Nevertheless, ‘business as usual’ will have very strong negative effects on local biodiversity through both climate and land-use effects, and mitigating both climate and land-use change will be essential to conserve local biodiversity.

## Supplementary Material

Supplementary Material

## Data accessibility

All the data used in this study are publicly available. The range maps for amphibians, mammals and reptiles are available at http://www.iucnredlist.org, and for birds at http://datazone.birdlife.org. The PREDICTS database describing land-use impacts was published previously as doi:10.5519/0066354.
