# Supplementary material for: Future effects of climate and land-use change on terrestrial vertebrate community diversity under different scenarios
Source: Proc Biol Sci. 2018 Jun 20;285(1881):20180792. doi: 10.1098/rspb.2018.0792 (PMC6030534; doi:10.1098/rspb.2018.0792)
Supplement: Supplementary Material [file rspb20180792supp1.pdf]

Detailed Methods

Models of climate impacts

To assess climate impacts on species, I use species distribution models, which relate estimates of species presence or absence in different locations to variables describing the environment [1]. Most applications of species distribution models to date have used point occurrence records of species. However, such records are strongly biased taxonomically and geographically (toward Western Europe, North America and Australasia in particular) [2,3]. Global estimates of climate-change impacts have therefore tended to use expert-drawn extent-of-occurrence distribution maps instead [4–6], and this is the approach that I adopt here. I show that predicted changes in the distributional area of species are consistent with previous global studies that have used point occurrence data (by comparison of Figure S6 with Figure 2 in ref. [7]). Distribution maps for amphibians, reptiles and mammals were obtained from IUCN [8], and for birds from BirdLife International [9]. I excluded from these raw range maps areas where species are considered vagrant, and areas where species are present only in the non-breeding season or during migration. I then further refined the range maps to exclude areas outside the known elevational limits of species, where available. Elevational limits were obtained from the website of the IUCN Red List (www.iucnredlist.org; accessed on 19th September 2016) for amphibians, reptiles and mammals, and from BirdLife International's World Bird Database (http://datazone.birdlife.org) for birds.

I used four climate variables, commonly reported to show a good association with vertebrate distributions [10]: minimum temperature of the coldest month, total annual precipitation, growing degree days, and water balance. I initially considered annual mean temperature as well, which has been used previously in applications of distribution models [10], but it showed a strong correlation globally with minimum temperature and growing degree days, which would have caused statistical problems for some of the modelling methods used (e.g. generalized linear models). Minimum temperature of the coldest month and total annual precipitation were taken directly from the WorldClim Version 1.4 dataset [11]. Growing degree days and water balance were calculated based on other climatic variables from WorldClim. Growing degree days was calculated using previously published methods [12] as follows. First, daily mean temperature values were interpolated from monthly values using cubic spline interpolation, implemented with the 'spline' function in R Version 3.3.2. Second, growing degree days was calculated as [12]:

$$GDD = \sum \max\{0, (T_m - T_t)\},$$

where T_m is the daily mean temperature and T_t is the threshold temperature above which growth is possible (assumed to be 5°C). I also calculated water balance using previously published methods, as the difference between total annual precipitation and total annual potential evapotranspiration (PET) [12]. Monthly PET values were calculated as [12]:

$$PET = 58.93 \times {T_{(above\ 0°C)}}/_{12},$$

where T is monthly average temperature. Monthly values were then summed to obtain total annual PET.

I resampled all of the distribution maps and climate data onto a 10-km × 10-km equal-area grid prior to analysis. All grid cells which intersected some part of a species' distribution polygon were considered to be occupied, to avoid very narrowly distributed species being discounted from the analysis. Climatic variables were resampled using bilinear interpolation.

I fit distribution models using five methods commonly used in other studies [1,13]: Maxent, Generalized Linear Models (GLM), Random Forests (RF), BIOCLIM and DOMAIN. I chose methods that allow the fitting of relatively simple functional forms, to avoid as much as possible overfitting the coarse-scale distribution maps used. Initially, I also explored the use of Generalized Additive Models (GAMs), which have been shown to produce an accurate representation of species' distributions [1], but these models proved to be computationally infeasible for generating global-scale models for all vertebrate species.

For all modelling approaches, I discounted all species that occupied fewer than 10 of the analysis grid cells. Upper limits on the number of occupied grid cells were also imposed for some of the modelling approaches, to maintain computational tractability (in terms of run-time and memory requirements): 400,000 for Maxent and GLM, 2,000 for RF, and 5,000 for BIOCLIM and DOMAIN. These limits were determined by experimentation of the time and memory requirements for the different models for different numbers of occupied grid cells.

Sampling of unoccupied grid cells was performed differently for the different modelling approaches. BIOCLIM and DOMAIN are presence-only approaches, not requiring information on unoccupied locations. For Maxent, 10,000 'background' points (sometimes fewer for very widespread species) were sampled randomly, without replacement, from among all grid cells (both occupied and unoccupied). For GLM and RF, a set of unoccupied cells, of equal number as the occupied cells (or fewer for very widespread species where the number of unoccupied grid cells available was smaller than the number of occupied grid cells), were sampled randomly without replacement [14]. As in previous studies [5], to avoid model over-fitting [14], the selection of background or pseudo-absence points was limited to realm-biome combinations occupied by the species' range map.

For Maxent and GLM models, the forms of the functions relating environmental variables to species' presence of absence can take a variety of complexities. To avoid overfitting, I restricted both models to use only linear and quadratic relationships. Random forests used the Breiman algorithm, with 500 candidate trees. BIOCLIM and DOMAIN are simple envelope approaches that fit species' distributions based on the area of climatic space encompassed by the occupied locations [1]. All distribution models were fit in R Version 3.3.2 [15]: GLM models were fit using the 'glm' function in the standard 'stats' package; Maxent, BIOCLIM and DOMAIN models were fit using their respective functions within the 'dismo' package Version 1.1-4 [16]; and RF models were fit using the 'randomForest' package Version 4.6-12 [17].

To evaluate the accuracy of each distribution model, I initially divided the dataset into 80% for calibrating the model and 20% for evaluating the model. This division was performed just once for each species-model combination, rather than performing a full cross-validation, because of the long time needed to run the models for all species. I evaluated all models using the Area Under the Receiver Operating Characteristic Curve (AUC) statistic. Although there are issues with using AUC values to assess distribution model accuracy [18], AUC is a simple, generally applicable measure of model accuracy, and is widely used in broad-scale applications of distribution models [5]. I considered models with an AUC value greater than 0.8 to be useful for making future projections. Models with this level of accuracy were generated for 20,932 species using Maxent, 20,932 using GLM, 20,938 using RF, 18,184 using BIOCLIM, and 17,876 using DOMAIN.

Models of land-use impacts

The models of land-use impacts followed previously published methods [19]. The models were based on data in the PREDICTS database [20], which contains data on the composition of assemblages in different land uses, drawn from individual publications from all over the

world. I used data for vertebrate species only from the 2016 public release of the PREDICTS database [21]. This subset of the database contained 479,642 records of the occurrence or abundance of 6,184 vertebrate species (20% of all described terrestrial vertebrate species [22]), from 158 publications [23–180], sampled at 7,585 locations spanning all terrestrial biomes except one (flooded grasslands and savannahs).

For this study, I modelled just one measure of local assemblage biodiversity: sampled species richness. Although measuring biodiversity only as species richness may not capture all important facets of biodiversity change [181], species richness remains a widely accepted and widely used metric [19]. Furthermore, there is no simple monotonic relationship between the predicted climatic suitability from species distribution models and other measures of biodiversity, such as abundance [182]. Species richness was modelled as a function of three measures of human pressure at each site: a broad categorization of land use, intensity of human land use and human population density. Land use was classified based on the description of the habitat given in the source publications using published criteria [183] into the following categories: primary vegetation for habitat never previously destroyed by human actions or extreme natural events, secondary vegetation for habitat recovering after prior destruction by human actions or extreme natural events (divided into young, intermediate and mature secondary vegetation depending on stage of recovery), plantation forest for areas planted with tree crops (e.g. fruit or timber), cropland for areas planted with herbaceous crops, pasture for areas grazed regularly or permanently by livestock, or urban for areas with human habitation or buildings. The intensity with which humans use the land was classified into three levels – minimal, light and intense – also using the description of the habitat given in the original source publications. The criteria used for classifying land-use intensity varied by land-use category. For example, intensity in primary vegetation was classified based on disturbances such as hunting or logging, whereas intensity in cropland was classified based on crop diversity, crop rotation, chemical inputs and degree of mechanization. The full set of criteria are published elsewhere [183]. Human population density was estimated by overlaying the sampled sites with published estimates of human population density [184] using Python code developed for the arcpy module of ArcMap Version 10.3 [185].

I modelled species richness as a function of the human pressure variables using generalized linear mixed-effects models [186]. These models were implemented using the glmmADMB package Version 0.8.3.3 [187] in R Version 3.2 [15]. I used a model with a negative binomial error distribution, to account for the over-dispersion present in sampled species richness values. I fit a random intercept of study identity to account for differences in broad location, sampling methods, sampling effort, and taxonomic group sampled among different published studies. All human pressure variables were fitted as fixed effects. For human population density, I considered linear and quadratic polynomial terms. I also considered interactions between land use and land-use intensity, and between land use and human population density. I selected the best-fitting combination of fixed-effects terms and their interactions using backward stepwise model selection. Interaction terms were tested first, and then dropped to test the main effects. Any main effects that were part of significant interaction terms were retained in the final model, regardless of their significance as main effects.

Climate and land-use scenarios

I applied my models of the response of biodiversity to climate and land use to the Representative Concentration Pathways (RCP) scenarios [188], the most recent global and widely used set of scenarios for which both climate and land-use estimates are available. There are four RCP scenarios, which make widely differing assumptions about future socio-

economic pathways and thus very different predictions about future climate and land use (see Table 1). The scenarios are labelled in terms of the radiative forcing (the amount by which the energy balance of the Earth is altered, in W/m^2) in 2100: 2.6, 4.5, 6.0 and 8.5. RCP 8.5 has been characterized as ‘business as usual’ [19,189] and most closely matches recent trends in greenhouse gas emissions [190].

I obtained climate estimates for the RCP scenarios from WorldClim Version 1.4 [11] at a spatial resolution of 5 arc-minutes. These data consist of averages across two future time periods 2041-2060 and 2061-2080. They were generated by applying predicted anomalies from global climate models (GCMs) to the WorldClim estimates of climate for the reference period (1960-1990). I obtained estimates from the 12 GCMs that produced estimates for all four scenarios: BCC-CSM1-1, CCSM4, GISS-E2-R, HadGEM2-AO, HadGEM2-ES, IPSL-CM5A-LR, MIROC-ESM-CHEM, MIROC-ESM, MIROC5, MRI-CGCM3, and NorESM1-M. The derived bioclimatic variables – growing degree days and water balance – were calculated in the same way as for the reference time period (see above) for each GCM and future time period. I then calculated the average (mean) predicted value for each climate variable and each future time period across all 12 GCMs. Finally, I projected all maps of future climate onto the same 10-km equal area grid as used for fitting the distribution models (see above), using a bilinear interpolation. When applying the distribution models onto the future climate estimates, I used only the multi-model means and not the prediction uncertainty across GCMs, for consistency with the land use projections (uncertainty estimates are not available for the RCP land-use estimates [191]).

Projections of land use were taken from the land-use harmonization project [192]. Land-use predictions are generated by a single model for each RCP scenario, precluding a consideration of model uncertainty in the predictions [191]. The land-use projections consist of estimates of the proportion of each terrestrial grid cell, at a spatial resolution of 0.5°, in each of 6 major land-use classes: primary vegetation, secondary vegetation, plantation forest, cropland, pasture and urban. Predictions are made for a single ‘secondary vegetation’ class. To divide this class according to stage of recovery – into young, intermediate and mature secondary vegetation – I used the estimates of the proportional area of each grid cell that will transition between different land-use classes each time step to calculate the age profile of secondary vegetation in each cell and for each time step, following the methods in ref. [19].

Directly associated projections of human population density are available for the historical land-use estimates, and for the RCP 4.5 and RCP 8.5 future scenarios. In the first and last of these cases, the projections consist of gridded estimates. For RCP 4.5, projections are made only at the country level. To resolve these country-level estimates into gridded spatial estimates, I assumed that spatial patterns within countries were constant and matched present-day patterns (using the population density estimates described above). For RCP 2.6 and RCP 6.0, as in ref. [19], I used country-level projections of human population density from the UN population projections. Specifically I used the ‘medium’ scenario, which most closely matches the global assumptions about human population density in the RCP 2.6 and RCP 6.0 scenarios. These country-level projections were resolved into gridded estimates using the same method as for the human population projections from RCP 4.5.

Estimates of land-use intensity, which is important in determining the effects of land use on biodiversity, are not directly available in the land-use estimates described above. Instead, as in ref. [19], I estimated land-use intensity for the present day, for the future and for the reference period using simple spatial models. To estimate current land-use intensity (in the year 2005), I used the map of ‘Global Land Systems’ [193] at a spatial resolution of 5 arc-minutes, which divides coarse land-use categories into finer divisions based on cropland intensity, pasture livestock density and human population density. Each global land system was assigned to one of the land use and land-use intensity combinations considered in the

models of the response of biodiversity [19]. Then the proportional area of each 0.5° × 0.5° grid cell occupied by each land use and land-use intensity combination was estimated as the proportion of finer 5-arc-minute cells within this coarser grid cell that had a corresponding global land system category. To project land-use intensity for the reference period (1990) and the future periods, I modelled land-use intensity in 2005 (using general linear models) as a function of the proportion of cell area occupied by its corresponding land use, and human population density, allowing relationships to vary among UN sub-regions. UN sub-region data came from the world borders shapefile version 0.3 (http://thematicmapping.org/downloads/world_borders.php), transformed into a raster at 0.5° spatial resolution using ArcMap version 10.3 [185]. These models were then applied onto land-use and human population density estimates for the reference and future periods, as described above, assuming the spatial distribution of UN sub-regions to be constant.

Projections of climate-impact models

All species distribution models were projected using the 'predict' function in the 'dismo' package Version 1.1-4 [16]. This produces estimates of the relative climatic suitability of each grid cell in the predicted area and for the predicted time period and scenario combination. However, the exact interpretation of this output varies depending on the modelling algorithm: sometimes values represent probability of presence, sometimes just relative suitability [194]. Therefore, it is common practice in projecting distribution models to convert the raw output to a binary prediction of species presence or absence. To do this, I used a threshold that minimizes the difference between model sensitivity and specificity, which has been shown generally to perform well [194].

I projected species distributions for the reference time period (1960-1990) and each of the two future time periods (2041-2060 and 2061-2080), and for each of the four RCP scenarios of climate change (see above). The distribution models were projected onto the future climate estimates at a spatial resolution of 5 arc-minutes, before being resampled (using bilinear interpolation) to 0.5° resolution to match the land-use projections. For each of the 12 scenario-time period combinations I made one of three assumptions about species ability to disperse in response to changing climatic suitability. For all dispersal scenarios, including the scenario of unlimited dispersal, I assumed that species could not move beyond the combination of biome and biogeographic realm in which they occurred in the reference time period, thus assuming that major habitats would not shift in response to climate changes within the time period simulated. Whether or not this assumption holds probably depends on the biome: on the one hand, trees still have not filled all potentially suitable areas following post-glacial climate changes [195]; on the other hand, desertification can occur very rapidly in response to climatic changes [196]. The least conservative dispersal scenario assumed unlimited dispersal ability, where a species was assumed to be able to occupy all climatically suitable areas (provided that they fell within an originally occupied realm-biome combination). The most conservative dispersal scenario assumed no ability of species to disperse in response to changing climatic suitability, with species only able to occupy areas that were suitable in the reference time period and that remained suitable in future time periods. Finally, I simulated an intermediate dispersal scenario (termed here, the 'realistic' scenario) assuming that species could move at a specified rate away from suitable areas in the reference time period, using clade-specific rates of dispersal: 0.5 km year^{-1} for reptiles and amphibians, and 3 km year^{-1} for mammals and birds. These rates correspond with the 'optimistic' clade-specific dispersal rates assumed in a previous study of vertebrate responses to climate change [7]. Specifically, a grid cell could be occupied in the future if its centroid fell within a certain distance of the nearest originally suitable cell, set according to the above

clade-specific dispersal rates. Dispersal rates vary substantially within these major clades of species [197–199], but species-specific estimates are not available for most vertebrate species.

Overall projections of species richness change were calculated for each grid cell by summing all of the species for which the cell was estimated to be climatically suitable, and then expressing this species richness as a percentage of the value for the same grid cell in the reference time period. Uncertainty in the climate projections was estimated as the full range of projected species richness values across all of the five model types in the ensemble.

Projections of land-use-impact models

The models of the response of vertebrate species richness to land use were projected onto the scenarios of land use, land-use intensity and human population density (see above) following previously published methods [19]. These projections assumed that the relative species richness of a given grid cell, as a percentage of the species richness estimated to occur naturally, is a simple multiplicative function of the proportional area and relative biodiversity values of the different land use and land-use intensity combinations within a grid cell. For example, if a cell were half covered by primary vegetation, which as the baseline land use has a species richness value of 100%, and half by intensively managed cropland, which has 65% of the species richness of primary vegetation, then the overall species richness estimate for the cell before accounting for the effect of human population density would be 82.5%. The effect of human population density was estimated assuming that humans were distributed uniformly throughout a grid cell. In reality, human population density probably co-varies with land use and land-use intensity, but the scenario data did not permit this level of detail in the projections. Uncertainty estimates (95% confidence intervals) for the projections of land-use impacts were derived from the uncertainty in the coefficients from the mixed-effects models of land-use responses (see above).

Combining the land-use and climate projections

The projections of land-use and climate impacts on local species richness were combined assuming that the pressures act on species independently of each other. For example, if a grid cell was predicted to retain 50% of its species as a result of climate impacts and 50% as a result of land-use impacts, then the resulting species richness value with the effect of both pressures would be 25%. This assumption is unlikely to hold for a number of reasons. First, climate and land use might disproportionately impact a similar set of species. For example, narrow-ranged species have been shown to be disproportionately sensitive to land use (Newbold et al., submitted), and are likely also to be the most sensitive to climate change. Second, climate has been shown – at least at small scales – to influence how species respond to land use [200,201]. Third, land use might influence the ability of species to disperse through landscapes in response to climate change [202]. At present there is insufficient information to account properly for these interactions, but they should be included in more refined models in future. I do however test whether climate and land use are likely to have the greatest effects on a similar set of species (see next section). Global averages for the combined projections of land-use and climate impacts (as well as for the projections of the effects of the pressures individually) were calculated as the average across all terrestrial grid cells, weighted by cell area and by total ‘natural’ vertebrate species richness, i.e. in the absence of any climate or land-use impacts. Total natural species richness was estimated by overlaying the extent-of-occurrence range maps – as described above – for all mammals, birds, reptiles and amphibians.

Supplementary Figures and Tables

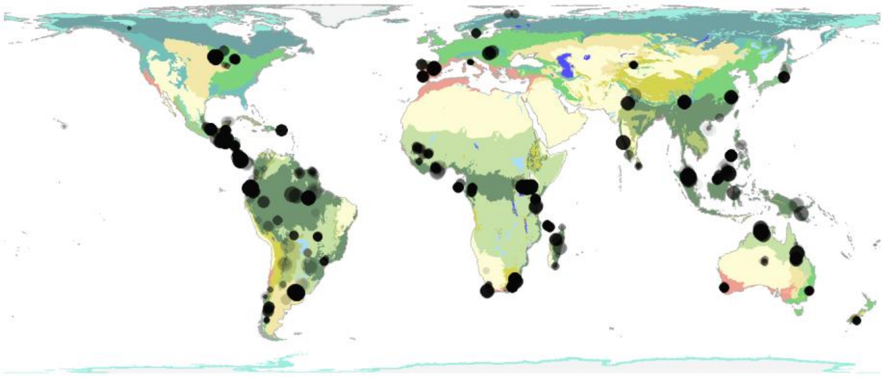

Figure S1. Map of sites with data used to assess the impact of land use on vertebrate community diversity. From the PREDICTS database [20,21]. Overlaid on a map of the world's terrestrial biomes [203]. Point diameter is proportional to the log-transformed number of species recorded at each site. All points have a fixed opacity, so areas that appear more opaque contain a greater number of sites.

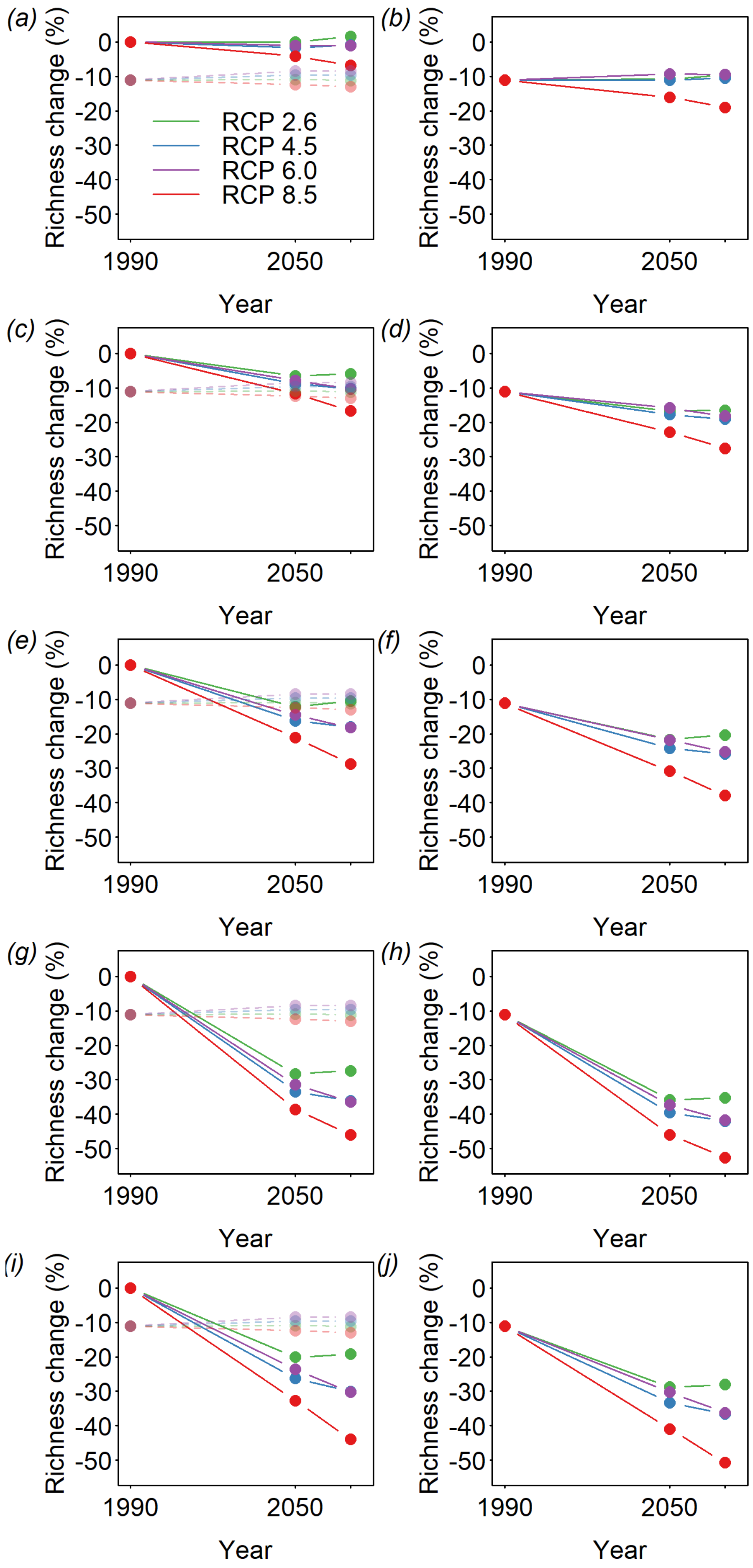

(a)
(b)
(c)
(d)
(e)
(f)
(g)
(h)
(i)
(j)
Richness change (%)
0
-10
-20
-30
-40
-50
1990
2050
Year
RCP 2.6
RCP 4.5
RCP 6.0
RCP 8.5

Figure S2. Predicted species richness of ecological communities under future climate and land-use change, for individual distribution modelling algorithms. Algorithms used were: Maxent (**a**, **b**), GLM (**c**, **d**), Random Forests (**e**, **f**), BIOCLIM (**g**, **h**) and DOMAIN (**i**, **j**). All values are expressed relative to a pre-human baseline. Left-hand panels (**a**, **c**, **e**, **g**, **i**) show separate effects of climate (solid, opaque lines) and land use (dashed, translucent lines); right-hand panels (**b**, **d**, **f**, **h**, **j**) show combined effects of both pressures (assuming no interactions).

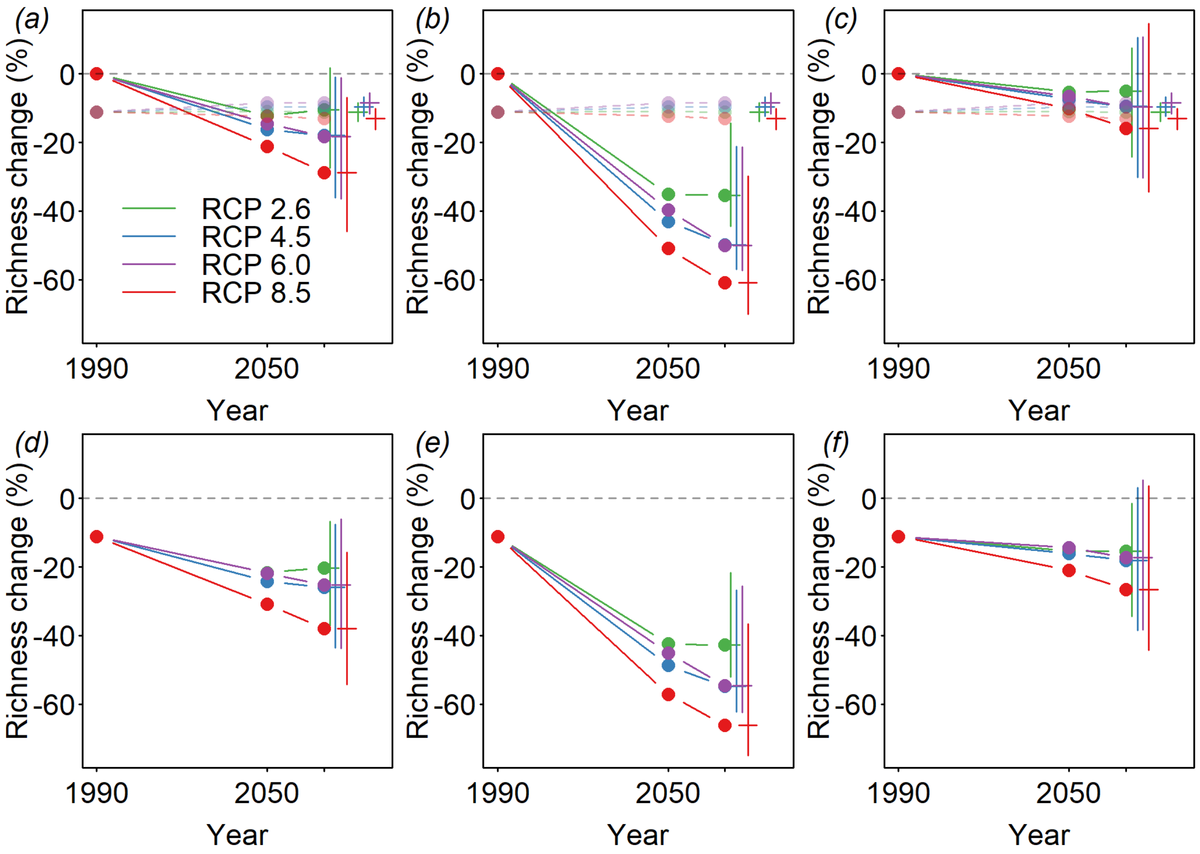

Figure S3. Predicted species richness of ecological communities under future climate and land-use change, for different dispersal assumptions. I assumed either clade-specific dispersal rates of 0.5 km year^{-1} for amphibians and reptiles and 3 km year^{-1} for birds and mammals (**a**, **d**); no ability to disperse beyond areas suitable in the baseline period (**b**, **e**); or unlimited dispersal ability (**c**, **f**). All values are expressed relative to a pre-human baseline. Upper panels (**a**, **b**, **c**) show separate effects of climate (solid, opaque lines) and land use (dashed, translucent lines); lower panels (**d**, **e**, **f**) show combined effects of both pressures (assuming no interactions). Error bars show estimated uncertainty in the projections for the year 2070: 95% confidence intervals for land-use impact models, and range of estimates across the distribution model ensemble for the climate impact models.

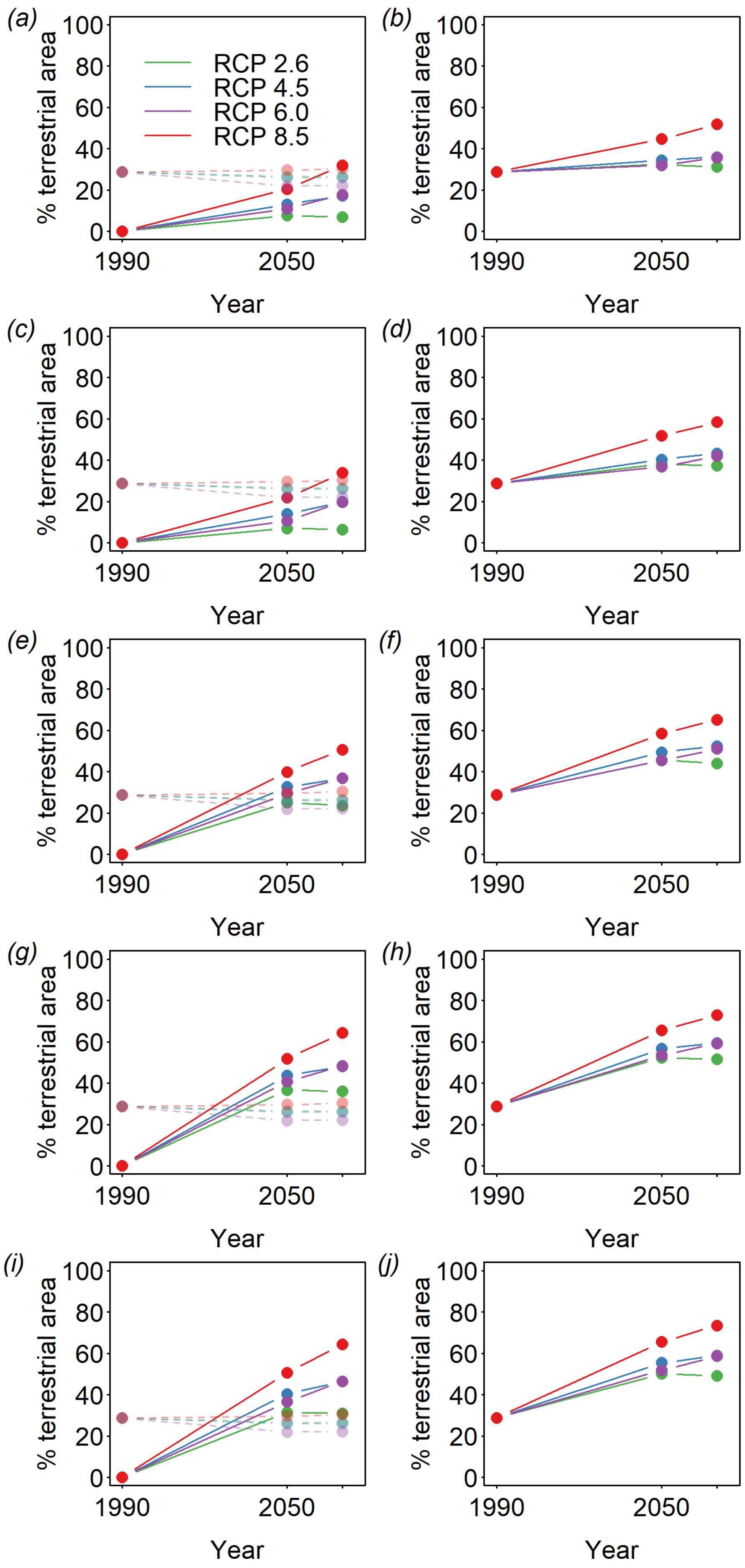

(a)
(b)
(c)
(d)
(e)
(f)
(g)
(h)
(i)
(j)
RCP 2.6
RCP 4.5
RCP 6.0
RCP 8.5
% terrestrial area
Year
1990
2050
0
20
40
60
80
100

Figure S4. Predicted proportion of the Earth's terrestrial surface exceeding 20% species loss under future climate and land-use change, for individual distribution modelling algorithms. Algorithms used were: Maxent (**a**, **b**), GLM (**c**, **d**), Random Forests (**e**, **f**), BIOCLIM (**g**, **h**) and DOMAIN (**i**, **j**). All values are expressed relative to a pre-human baseline. Lefthand panels (**a**, **c**, **e**, **g**, **i**) show separate effects of climate (solid, opaque lines) and land use (dashed, translucent lines); right-hand panels (**b**, **d**, **f**, **h**, **j**) show combined effects of both pressures (assuming no interactions).

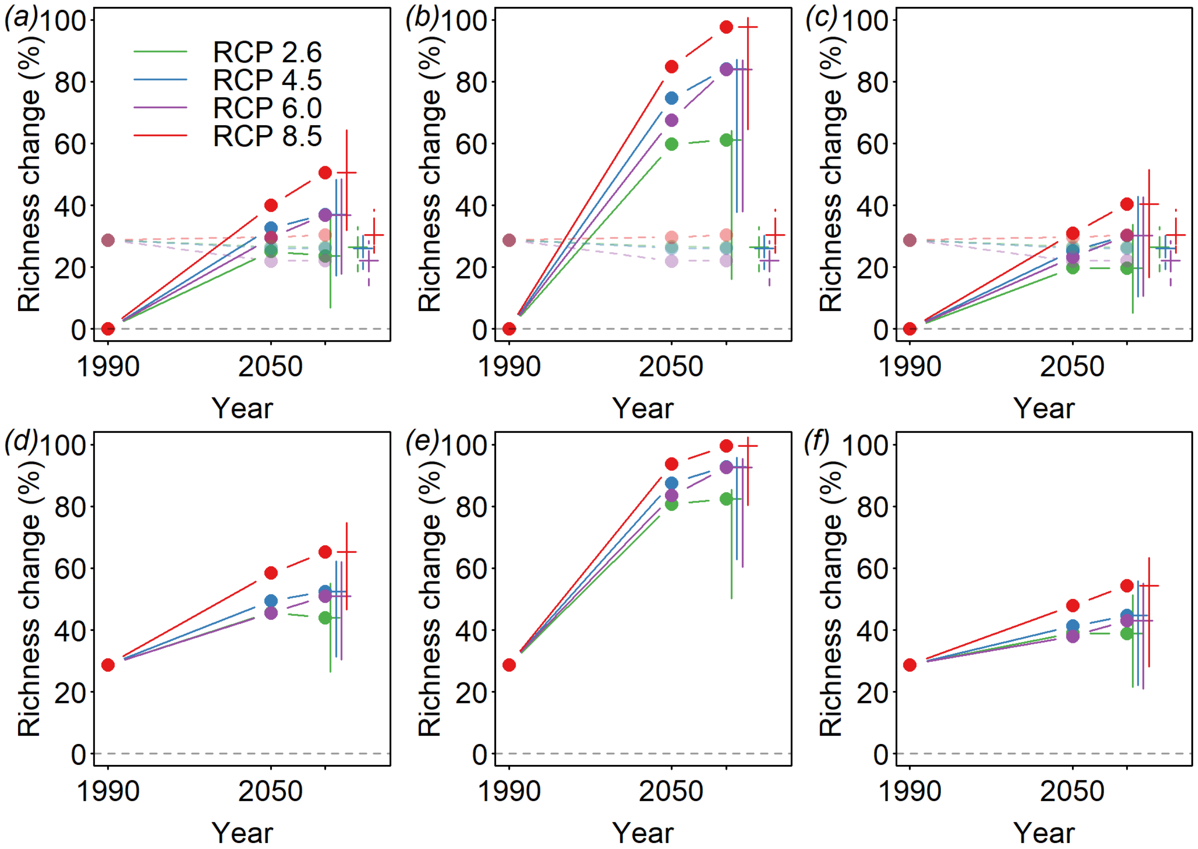

Figure S5. Predicted proportion of the Earth's terrestrial surface exceeding 20% species loss under future climate and land-use change, for different dispersal assumptions. I assumed either clade-specific dispersal rates of 0.5 km year^{-1} for amphibians and reptiles and 3 km year^{-1} for birds and mammals (**a**, **d**); no ability to disperse beyond areas suitable in the baseline period (**b**, **e**); or unlimited dispersal ability (**c**, **f**). All values are expressed relative to a pre-human baseline. Upper panels (**a**, **b**, **c**) show separate effects of climate (solid, opaque lines) and land use (dashed, translucent lines); lower panels (**d**, **e**, **f**) show combined effects of both pressures (assuming no interactions). Error bars show estimated uncertainty in the projections for the year 2070: 95% confidence intervals for land-use impact models, and range of estimates across the distribution model ensemble for the climate impact models.

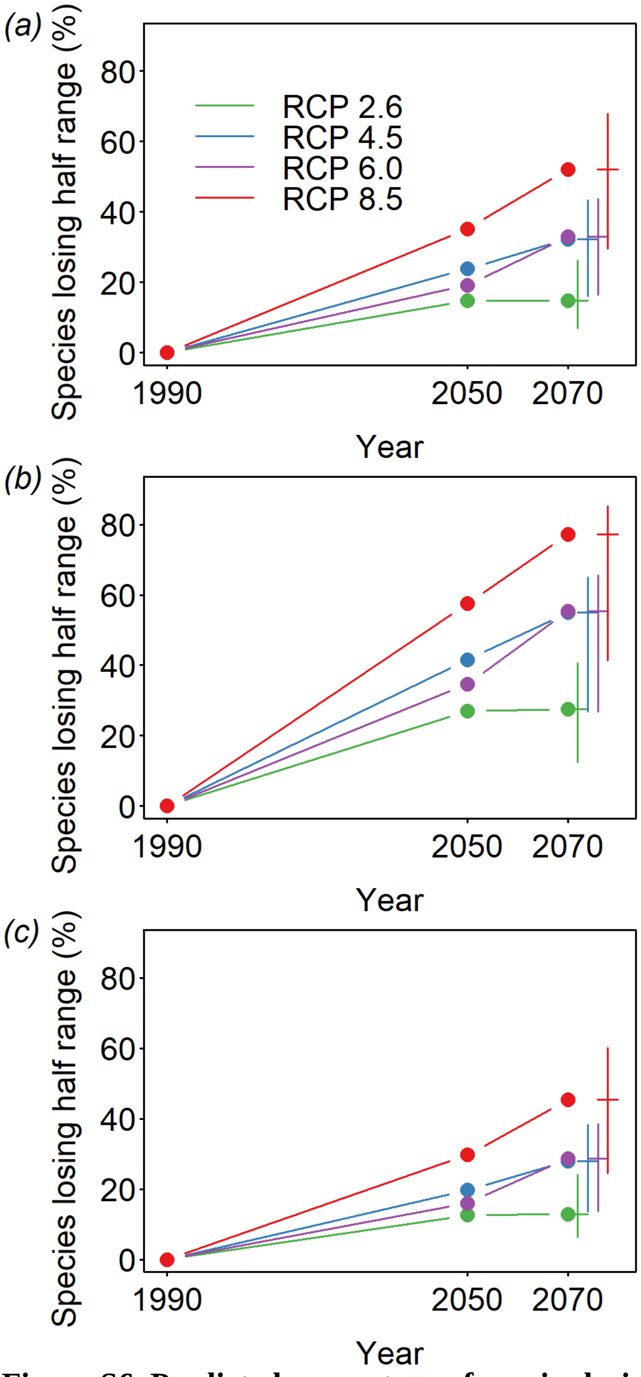

Figure S6. Predicted percentage of species losing more than half of their distribution area under future climate change, for different dispersal assumptions. I assumed either clade-specific dispersal rates of 0.5 km year^{-1} for amphibians and reptiles and 3 km year^{-1} for birds and mammals (**a**); no ability to disperse beyond areas suitable in the baseline period (**b**); or unlimited dispersal ability (**c**). All values are expressed relative to the baseline period (1960-1990). Error bars show estimated uncertainty in the projections for the year 2070, as the range of estimates across the distribution model ensemble. A comparison of the results in **a** with Figure 2 in ref. [7] shows broad consistency, although note that slightly different climate scenarios were used.

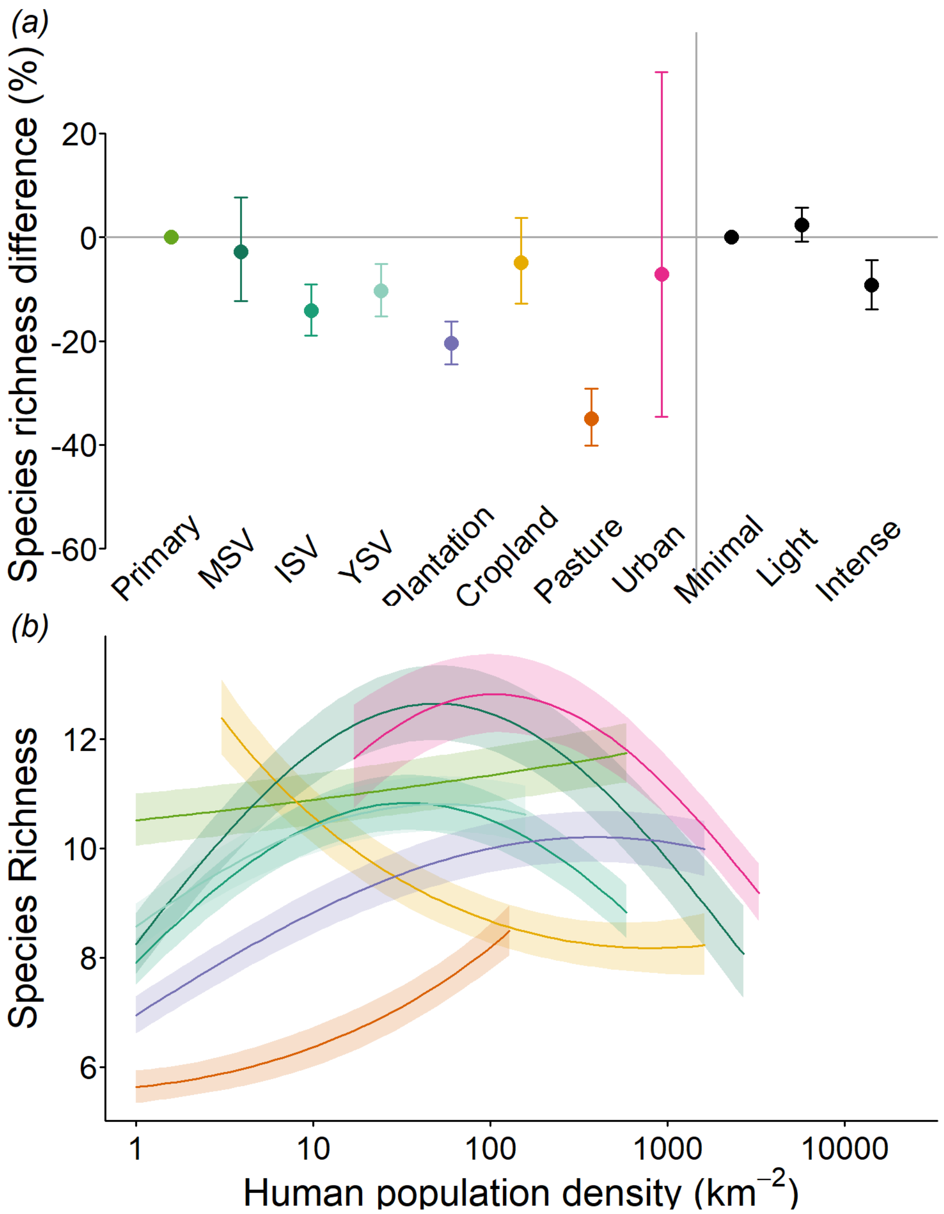

Figure S7. Response of local species richness to land use and land-use intensity (a) and human population density (b). Species richness in **a** is shown as a percentage change relative to the baseline condition (primary vegetation), with error bars showing 95% confidence intervals. Species richness in **b** is shown as absolute expected average species richness at each level of human population density and for each land use, with shading showing ±0.5 × SEM, for clarity. Colours used for each land use are the same in **b** as in **a**.

Table S1. Summary of predictions about climate, land use and human population made by the Representative Concentration Pathways Scenarios. Adapted from Table 1 in ref. [19]. Land use and human population estimates are from ref. [188], and climate from ref. [204]. The scenarios are labelled in terms of the radiative forcing (the amount by which the energy balance of the Earth is altered, in W/m^2) in 2100: 2.6, 4.5, 6.0 and 8.5.

**Scenario**	**Climate in 2070**	**Land use**	**Human population**
RCP 2.6	Approximately 1°C.	Shift in agriculture from developed to developing countries. Large expansion of biofuel plantations. Urban area assumed to be constant.	10.1 billion by 2100.
RCP 4.5	Approximately 1.6°C.	Carbon markets lead to slower loss of primary forest and rapid expansion of secondary forest, especially in the tropics. Improved crop yields and agricultural efficiency, as well as dietary changes, cause decreases in agricultural area. Urban area assumed to be constant.	Reaches peak at 9 billion around 2065.
RCP 6.0	Approximately 1.6°C.	Human population growth drives an increase in cropland and urban areas. Decline in livestock grazing areas (pastures).	9.1 billion by 2100.
RCP 8.5	Approximately 2.7°C.	Agricultural areas expand to feed a growing human population, despite yield increases. Urban areas expand to house the human population.	12 billion by 2100.

Supplementary References

1. Elith J *et al.* 2006 Novel methods improve prediction of species' distributions from occurrence data. *Ecography* **29**, 129–151. (doi:10.1111/j.2006.0906-7590.04596.x)
2. Newbold T. 2010 Applications and limitations of museum data for conservation and ecology, with particular attention to species distribution models. *Prog. Phys. Geogr.* **34**, 3–22. (doi:10.1177/0309133309355630)
3. Meyer C, Kreft H, Guralnick R, Jetz W. 2015 Global priorities for an effective information basis of biodiversity distributions. *Nat. Commun.* **6**, 8221. (doi:10.1038/ncomms9221)
4. Lawler JJ, Shafer SL, White D, Kareiva P, Maurer EP, Blaustein AR, Bartlein PJ. 2009 Projected climate-induced faunal change in the Western Hemisphere. *Ecology* **90**, 588–597.
5. Visconti P *et al.* 2016 Projecting global biodiversity indicators under future development scenarios. *Conserv. Lett.* **9**, 5–13. (doi:10.1111/conl.12159)
6. Jetz W, Wilcove DS, Dobson AP. 2007 Projected impacts of climate and land-use change on the global diversity of birds. *PLoS Biol.* **5**, e157. (doi:10.1371/journal.pbio.0050157)
7. Warren R *et al.* 2013 Quantifying the benefit of early climate change mitigation in avoiding biodiversity loss. *Nat. Clim. Chang.* **3**, 678–682. (doi:10.1038/nclimate1887)
8. IUCN. 2013 *The IUCN Red List of Threatened Species. Version 2013.7*. See: http://www.iucnredlist.org.
9. Birdlife International, NatureServe. 2012 *Bird species distribution maps of the world. Version 2.0*. See: http://www.birdlife.org/datazone/info/spcdownload.
10. Araújo MB, Alagador D, Cabeza M, Nogués-Bravo D, Thuiller W. 2011 Climate change threatens European conservation areas. *Ecol. Lett.* **14**, 484–492. (doi:10.1111/j.1461-0248.2011.01610.x)
11. Hijmans RJ, Cameron SE, Parra JL, Jones PG, Jarvis A. 2005 Very high resolution interpolated climate surfaces for global land areas. *Int. J. Climatol.* **25**, 1965–1978. (doi:10.1002/joc.1276)
12. Skov F, Svenning J-C. 2004 Potential impact of climatic change on the distribution of forest herbs in Europe. *Ecography* **27**, 366–380. (doi:10.1111/j.0906-7590.2004.03823.x)
13. Marmion M, Parviainen M, Luoto M, Heikkinen RK, Thuiller W. 2009 Evaluation of consensus methods in predictive species distribution modelling. *Divers. Distrib.* **15**, 59–69. (doi:10.1111/j.1472-4642.2008.00491.x)
14. Barbet-Massin M, Jiguet F, Albert CH, Thuiller W. 2012 Selecting pseudo-absences for species distribution models: how, where and how many? *Methods Ecol. Evol.* **3**, 327–338. (doi:10.1111/j.2041-210X.2011.00172.x)
15. R Core Team. 2016 *R: A Language and Environment for Statistical Computing*. See: https://cran.r-project.org.
16. Hijmans RJ, Phillips S, Leathwick J, Elith J. 2017 *dismo: Species Distribution Modeling. R package version 1.1-4*. See: https://CRAN.R-project.org/package=dismo.
17. Liaw A, Wiener M. 2002 Classification and Regression by randomForest. *R News* **2**, 18–22.
18. Lobo JM, Jiménez-Valverde A, Real R. 2008 AUC: a misleading measure of the performance of predictive distribution models. *Glob. Ecol. Biogeogr.* **17**, 145–151. (doi:10.1111/j.1466-8238.2007.00358.x)
19. Newbold T *et al.* 2015 Global effects of land use on local terrestrial biodiversity. *Nature* **520**, 45–50. (doi:10.1038/nature14324)

20. Hudson LN *et al.* 2017 The database of the PREDICTS (Projecting Responses of Ecological Diversity In Changing Terrestrial Systems) project. *Ecol. Evol.* **7**, 145–188. (doi:10.1002/ece3.2579)
21. Hudson LN *et al.* 2016 *Dataset: The 2016 release of the PREDICTS database*. (doi:10.5519/0066354). See: http://data.nhm.ac.uk/dataset/902f084d-ce3f-429f-a6a5-23162c73fdf7.
22. Chapman AD. 2009 *Numbers of Living Species in Australia and the World*. Australian Biological Resources Study, Canberra, Australia.
23. Aben J, Dorenbosch M, Herzog SK, Smolders AJP, Van Der Velde G. 2008 Human disturbance affects a deciduous forest bird community in the Andean foothills of central Bolivia. *Bird Conserv. Int.* **18**, 363–380. (doi:10.1017/s0959270908007326)
24. Adum GB, Eichhorn MP, Oduro W, Ofori-Boateng C, Rodel MO. 2013 Two-stage recovery of amphibian assemblages following selective logging of tropical forests. *Conserv. Biol.* **27**, 354–363. (doi:10.1111/cobi.12006)
25. Alcala EL, Alcala a. C, Dolino CN. 2004 Amphibians and reptiles in tropical rainforest fragments on Negros Island, the Philippines. *Environ. Conserv.* **31**, 254–261. (doi:10.1017/S0376892904001407)
26. Arbeláez-Cortés E, Rodríguez-Correa HA, Restrepo-Chica M. 2011 Mixed bird flocks: patterns of activity and species composition in a region of the Central Andes of Colombia. *Rev. Mex. Biodivers.* **82**, 639–651.
27. Aumann T. 2001 The structure of raptor assemblages in riparian environments in the south-west of the Northern Territory, Australia. *Emu* **101**, 293–304. (doi:10.1071/mu00072)
28. Azhar B, Lindenmayer DB, Wood J, Fischer J, Manning A, Mcelhinny C, Zakaria M. 2013 The influence of agricultural system, stand structural complexity and landscape context on foraging birds in oil palm landscapes. *Ibis* **155**, 297–312.
29. Azman NM, Latip NSA, Sah SAM, Akil MAMM, Shafie NJ, Khairuddin NL. 2011 Avian diversity and feeding guilds in a secondary forest, an oil palm plantation and a paddy field in Riparian areas of the Kerian River Basin, Perak, Malaysia. *Trop. Life Sci. Res.* **22**, 45–64.
30. Azpiroz AB, Blake JG. 2009 Avian assemblages in altered and natural grasslands in the northern Campos of Uruguay. *Condor* **111**, 21–35. (doi:10.1525/cond.2009.080111)
31. Báldi A, Batáry P, Erdős S. 2005 Effects of grazing intensity on bird assemblages and populations of Hungarian grasslands. *Agric. Ecosyst. Environ.* **108**, 251–263. (doi:10.1016/j.agee.2005.02.006)
32. Barlow J *et al.* 2007 Quantifying the biodiversity value of tropical primary, secondary, and plantation forests. *Proc. Natl. Acad. Sci. U. S. A.* **104**, 18555–18560. (doi:10.1073/pnas.0703333104)
33. Barlow J, Mestre LAM, Gardner TA, Peres CA. 2007 The value of primary, secondary and plantation forests for Amazonian birds. *Biol. Conserv.* **136**, 212–231. (doi:10.1016/j.biocon.2006.11.021)
34. Bartolommei P, Mortelliti A, Pezzo F, Puglisi L. 2013 Distribution of nocturnal birds (Strigiformes and Caprimulgidae) in relation to land-use types, extent and configuration in agricultural landscapes of Central Italy. *Rend. Lincei-Scienze Fis. E Nat.* **24**, 13–21. (doi:10.1007/s12210-012-0211-3)
35. Bernard H, Fjeldsa J, Mohamed M. 2009 A case study on the effects of disturbance and conversion of tropical lowland rain forest on the non-volant small mammals in north Borneo: management implications. *Mammal Study* **34**, 85–96. (doi:10.3106/041.034.0204)

36. Bicknell J, Peres CA. 2010 Vertebrate population responses to reduced-impact logging in a neotropical forest. *For. Ecol. Manage.* **259**, 2267–2275. (doi:10.1016/j.foreco.2010.02.027)
37. Bóçon R. 2010 *Riqueza e abundância de aves em três estágios sucessionais da floresta ombrófila densa submontana, Antonina, Paraná*. PhD Thesis, Hungarian Academy of Sciences
38. Borges SH. 2007 Bird assemblages in secondary forests developing after slash-and-burn agriculture in the Brazilian Amazon. *J. Trop. Ecol.* **23**, 469–477. (doi:10.1017/s0266467407004105)
39. Brandt JS, Wood EM, Pidgeon AM, Han L-X, Fang Z, Radeloff VC. 2013 Sacred forests are keystone structures for forest bird conservation in southwest China's Himalayan Mountains. *Biol. Conserv.* **166**, 34–42. (doi:10.1016/j.biocon.2013.06.014)
40. Cáceres NC, Nápoli RP, Casella J, Hannibal W. 2010 Mammals in a fragmented savannah landscape in south-western Brazil. *J. Nat. Hist.* **44**, 491–512. (doi:10.1080/00222930903477768)
41. Cagle NL. 2008 Snake species distributions and temperate grasslands: a case study from the American tallgrass prairie. *Biol. Conserv.* **141**, 744–755. (doi:10.1016/j.biocon.2008.01.003)
42. Cassano C. 2014 Forest loss or management intensification? Identifying causes of mammal decline in cacao agroforests. **169**, 14–22. (doi:10.1016/j.biocon.2013.10.006)
43. Castro-Luna AA, Sosa VJ, Castillo-Campos G. 2007 Bat diversity and abundance associated with the degree of secondary succession in a tropical forest mosaic in south-eastern Mexico. *Anim. Conserv.* **10**, 219–228. (doi:10.1111/j.1469-1795.2007.00097.x)
44. Centro Agronómico Tropical de Investigación y Enseñanza. 2010 *Unpublished data of reptilian and amphibian diversity in six countries in Central America*.
45. Cerezo A, Conde MC, Poggio SL. 2011 Pasture area and landscape heterogeneity are key determinants of bird diversity in intensively managed farmland. *Biodivers. Conserv.* **20**, 2649–2667. (doi:10.1007/s10531-011-0096-y)
46. Chapman KA, Reich PB. 2007 Land use and habitat gradients determine bird community diversity and abundance in suburban, rural and reserve landscapes of Minnesota, USA. *Biol. Conserv.* **135**, 527–541. (doi:10.1016/j.biocon.2006.10.050)
47. Clarke FM, Rostant L V, Racey PA. 2005 Life after logging: post-logging recovery of a neotropical bat community. *J. Appl. Ecol.* **42**, 409–420. (doi:10.1111/j.1365-2664.2005.01024.x)
48. Cockle KL, Leonard ML, Bodrati AA. 2005 Presence and abundance of birds in an Atlantic forest reserve and adjacent plantation of shade-grown yerba mate, in Paraguay. *Biodivers. Conserv.* **14**, 3265–3288. (doi:10.1007/s10531-004-0446-0)
49. Craig MD, Grigg AH, Garkaklis MJ, Hobbs RJ, Grant CD, Fleming PA, Hardy GESJ. 2009 Does habitat structure influence capture probabilities? A study of reptiles in a eucalypt forest. *Wildl. Res.* **36**, 509–515. (doi:10.1071/wr09014)
50. Craig MD, Grigg AH, Hobbs RJ, Hardy GESJ. 2014 Does coarse woody debris density and volume influence the terrestrial vertebrate community in restored bauxite mines? *For. Ecol. Manage.* **318**, 142–150. (doi:10.1016/j.foreco.2014.01.011)
51. Craig MD, Hardy GESJ, Fontaine JB, Garkakalis MJ, Grigg AH, Grant CD, Fleming PA, Hobbs RJ. 2012 Identifying unidirectional and dynamic habitat filters to faunal recolonisation in restored mine-pits. *J. Appl. Ecol.* **49**, 919–928. (doi:10.1111/j.1365-2664.2012.02152.x)
52. Craig MD, Stokes VL, Hardy GES, Hobbs RJ. 2015 Edge effects across boundaries between natural and restored jarrah (*Eucalyptus marginata*) forests in south-western Australia. *Austral Ecol.* **40**, 186–197. (doi:10.1111/aec.12193)

53. Dallimer M, Parnell M, Bicknell JE, Melo M. 2012 The importance of novel and agricultural habitats for the avifauna of an oceanic island. *J. Nat. Conserv.* **20**, 191–199. (doi:10.1016/j.jnc.2012.04.001)
54. Danquah E, Oppong SK, Nutsuakor ME. 2012 Effect of protected area category on mammal abundance in Western Ghana. *J. Biodivers. Environ. Sci.* **2**, 50–57.
55. Dawson J, Turner C, Pileng O, Farmer A, McGary C, Walsh C, Tamblyn A, Yosi C. 2011 Bird communities of the lower Waria Valley, Morobe Province, Papua New Guinea: a comparison between habitat types. *Trop. Conserv. Sci.* **4**, 317–348.
56. D'Cruze N, Kumar S. 2011 Effects of anthropogenic activities on lizard communities in northern Madagascar. *Anim. Conserv.* **14**, 542–552.
57. de Lima RF, Dallimer M, Atkinson PW, Barlow J. 2013 Biodiversity and land-use change: understanding the complex responses of an endemic-rich bird assemblage. *Divers. Distrib.* **19**, 411–422. (doi:10.1111/ddi.12015)
58. de Souza VM, de Souza B, Morato EF. 2008 Effect of the forest succession on the anurans (Amphibia: Anura) of the Reserve Catuaba and its periphery, Acre, southwestern Amazonia. *Rev. Bras. Zool.* **25**, 49–57.
59. de Thoisy B, Richard-Hansen C, Goguillon B, Joubert P, Obstancias J, Winterton P, Brosse S. 2010 Rapid evaluation of threats to biodiversity: human footprint score and large vertebrate species responses in French Guiana. *Biodivers. Conserv.* **19**, 1567–1584. (doi:10.1007/s10531-010-9787-z)
60. Doulton H, Marsh C, Newman A, Bird K, Bell M. 2007 *Conservation Comores 2005: biodiversity and resource-use assessment and environmental awareness*. University of Oxford, Oxford, UK.
61. Dures SG, Cumming GS. 2010 The confounding influence of homogenising invasive species in a globally endangered and largely urban biome: Does habitat quality dominate avian biodiversity? *Biol. Conserv.* **143**, 768–777. (doi:10.1016/j.biocon.2009.12.019)
62. Edenius L, Mikusinski G, Bergh J. 2011 Can repeated fertilizer applications to young Norway spruce enhance avian diversity in intensively managed forests? *Ambio* **40**, 521–527. (doi:10.1007/s13280-011-0137-5)
63. Endo W, Peres CA, Salas E, Mori S, Sanchez-Vega JL, Shepard GH, Pacheco V, Yu DW. 2010 Game Vertebrate Densities in Hunted and Nonhunted Forest Sites in Manu National Park, Peru. *Biotropica* **42**, 251–261. (doi:10.1111/j.1744-7429.2009.00546.x)
64. Fabricius C, Burger M, Hockey PAR. 2003 Comparing biodiversity between protected areas and adjacent rangeland in xeric succulent thicket, South Africa: arthropods and reptiles. *J. Appl. Ecol.* **40**, 392–403. (doi:10.1046/j.1365-2664.2003.00793.x)
65. Faruk A, Belabut D, Ahmad N, Knell RJ, Garner TWJ. 2013 Effects of oil-palm plantations on diversity of tropical anurans. *Conserv. Biol.* **27**, 615–624. (doi:10.1111/cobi.12062)
66. Farwig N, Sajita N, Böhning-Gaese K. 2008 Conservation value of forest plantations for bird communities in western Kenya. *For. Ecol. Manage.* **255**, 3885–3892. (doi:10.1016/j.foreco.2008.03.042)
67. Fernandez IC, Simonetti JA. 2013 Small mammal assemblages in fragmented shrublands of urban areas of Central Chile. *Urban Ecosyst.* **16**, 377–387. (doi:10.1007/s11252-012-0272-1)
68. Flaspohler DJ, Giardina CP, Asner GP, Hart P, Price J, Lyons CK, Castaneda X. 2010 Long-term effects of fragmentation and fragment properties on bird species richness in Hawaiian forests. *Biol. Conserv.* **143**, 280–288. (doi:10.1016/j.biocon.2009.10.009)
69. Fukuda D, Tisen OB, Momose K, Sakai S. 2009 Bat diversity in the vegetation mosaic around a lowland dipterocarp forest of Borneo. *Raffles Bull. Zool.* **57**, 213–221.

70. Furlani D, Ficetola GF, Colombo G, Ugurlucan M, De Bernardi F. 2009 Deforestation and the structure of frog communities in the Humedale Terraba-Sierpe, Costa Rica. *Zoolog. Sci.* **26**, 197–202. (doi:10.2108/zsj.26.197)
71. García KP, Ortiz Zapata JC, Aguayo M, D'Elia G. 2013 Assessing rodent community responses in disturbed environments of the Chilean Patagonia. *Mammalia* **77**, 195–204. (doi:10.1515/mammalia-2011-0134)
72. García-R JC, Cárdenas-H H, Castro-H F. 2007 Relationship between anurans diversity and successional stages of a very humid low montane forest in Valle del Cauca, southwestern of Colombia. *Caldasia* **29**, 363–374.
73. Garmendia A, Arroyo-Rodriguez V, Estrada A, Naranjo EJ, Stoner KE. 2013 Landscape and patch attributes impacting medium- and large-sized terrestrial mammals in a fragmented rain forest. *J. Trop. Ecol.* **29**, 331–344. (doi:10.1017/s0266467413000370)
74. Gheler-Costa C, Vettorazzi CA, Pardini R, Verdade LM. 2012 The distribution and abundance of small mammals in agroecosystems of southeastern Brazil. *Mammalia* **76**, 185–191. (doi:10.1515/mammalia-2011-0109)
75. Gomes LGL, Oostra V, Nijman V, Cleef AM, Kappelle M. 2008 Tolerance of frugivorous birds to habitat disturbance in a tropical cloud forest. *Biol. Conserv.* **141**, 860–871. (doi:10.1016/j.biocon.2008.01.007)
76. Granjon L, Duplantier JM. 2011 Guinean biodiversity at the edge: rodents in forest patches of southern Mali. *Mamm. Biol.* **76**, 583–591. (doi:10.1016/j.mambio.2011.06.003)
77. Gutierrez-Lamus DL. 2004 Composition and abundance of Anura in two forest types (natural and planted) in the eastern Cordillera of Colombia. *Caldasia* **26**, 245–264.
78. Hassan SN, Salum AR, Rija AA, Modest R, Kideghesho R. J, Malata PF. 2013 Human-induced disturbances influence on bird communities of coastal forests in Eastern Tanzania. *Br. J. Appl. Sci. Technol.* **3**, 48–64. (doi:10.9734/bjast/2014/2200)
79. Hayward MW. 2009 Bushmeat hunting in Dwesa and Cwebe nature reserves, Eastern Cape, South Africa. *South African J. Wildl. Res.* **39**, 70–84. (doi:10.3957/056.039.0108)
80. Henschel P. 2008 *The Conservation biology of the leopard Panthera pardus in Gabon: status, threats and strategies for conservation*. PhD Thesis, Georg-August-Universität Göttingen.
81. Herrera JP, Wright PC, Lauterbur E, Ratovonjanahary L, Taylor LL. 2011 The effects of habitat disturbance on lemurs at Ranomafana National Park, Madagascar. *Int. J. Primatol.* **32**, 1091–1108. (doi:10.1007/s10764-011-9525-8)
82. Hilje B, Aide TM. 2012 Recovery of amphibian species richness and composition in a chronosequence of secondary forests, northeastern Costa Rica. *Biol. Conserv.* **146**, 170–176. (doi:10.1016/j.biocon.2011.12.007)
83. Hoffmann A, Zeller U. 2005 Influence of variations in land use intensity on species diversity and abundance of small mammals in the Nama Karoo, Namibia. *Belgian J. Zool.* **135**, 91–96.
84. Ims RA, Henden JA. 2012 Collapse of an arctic bird community resulting from ungulate-induced loss of erect shrubs. *Biol. Conserv.* **149**, 2–5. (doi:10.1016/j.biocon.2012.02.008)
85. Isaacs-Cubides PJ, Urbina-Cardona JN. 2011 Anthropogenic disturbance and edge effects on anuran assemblages inhabiting cloud forest fragments in Colombia. *Nat. Conserv.* **9**, 39–46. (doi:10.4322/natcon.2011.004)
86. Jolli V, Pandit MK. 2011 Monitoring pheasants (Phasianidae) in the western Himalayas to measure the impact of hydro-electric projects. *Ring* **33**, 37–46.

(doi:10.2478/v10050-011-0003-7)
87. Jung TS, Powell T. 2011 Spatial distribution of meadow jumping mice (*Zapus hudsonius*) in logged boreal forest of northwestern Canada. *Mamm. Biol.* **76**, 678–682. (doi:10.1016/j.mambio.2011.08.002)
88. Kessler M *et al.* 2009 Alpha and beta diversity of plants and animals along a tropical land-use gradient. *Ecol. Appl.* **19**, 2142–56.
89. Kittle AM, Watson AC, Chanaka Kumara PH, Nimalka Sanjeewani HK. 2012 Status and distribution of the leopard in the central hills of Sri Lanka. *Cat News* **56**, 28–31.
90. Kurz DJ, Nowakowski AJ, Tingley MW, Donnelly MA, Wilcove DS. 2014 Forest-land use complementarity modifies community structure of a tropical herpetofauna. *Biol. Conserv.* **170**, 246–255. (doi:10.1016/j.biocon.2013.12.027)
91. Kutt AS, Vanderduys EP, O'Reagain P. 2012 Spatial and temporal effects of grazing management and rainfall on the vertebrate fauna of a tropical savanna. *Rangel. J.* **34**, 173–182. (doi:10.1071/rj11049)
92. Kutt AS, Woinarski JCZ. 2007 The effects of grazing and fire on vegetation and the vertebrate assemblage in a tropical savanna woodland in north-eastern Australia. *J. Trop. Ecol.* **23**, 95–106. (doi:10.1017/s0266467406003579)
93. Lantschner M V, Rusch V, Hayes JP. 2012 Habitat use by carnivores at different spatial scales in a plantation forest landscape in Patagonia, Argentina. *For. Ecol. Manage.* **269**, 271–278. (doi:10.1016/j.foreco.2011.12.045)
94. Lantschner M V, Rusch V, Peyrou C. 2008 Bird assemblages in pine plantations replacing native ecosystems in NW Patagonia. *Biodivers. Conserv.* **17**, 969–989. (doi:10.1007/s10531-007-9243-x)
95. Lasky JR, Keitt TH. 2010 Abundance of Panamanian dry-forest birds along gradients of forest cover at multiple scales. *J. Trop. Ecol.* **26**, 67–78. (doi:10.1017/s0266467409990368)
96. Latta SC, Tinoco BA, Astudillo PX, Graham CH. 2011 Patterns and magnitude of temporal change in avian communities in the Ecuadorian Andes. *Condor* **113**, 24–40. (doi:10.1525/cond.2011.090252)
97. Laurance WF, Laurance SGW. 1996 Responses of five arboreal marsupials to recent selective logging in tropical Australia. *Biotropica* **28**, 310–322. (doi:10.2307/2389195)
98. Lehouck V, Spanhove T, Colson L, Adringa-Davis A, Cordeiro NJ, Lens L. 2009 Habitat disturbance reduces seed dispersal of a forest interior tree in a fragmented African cloud forest. *Oikos* **118**, 1023–1034. (doi:10.1111/j.1600-0706.2009.17300.x)
99. Li SN, Zou FS, Zhang Q, Sheldon FH. 2013 Species richness and guild composition in rubber plantations compared to secondary forest on Hainan Island, China. *Agrofor. Syst.* **87**, 1117–1128. (doi:10.1007/s10457-013-9624-y)
100. Luja VH, Herrando-Perez S, Gonzalez-Solis D, Luiselli L. 2008 Secondary rain forests are not havens for reptile species in tropical Mexico. *Biotropica* **40**, 747–757. (doi:10.1111/j.1744-7429.2008.00439.x)
101. Macip-Ríos R, Muñoz-Alonso A. 2008 Diversidad de lagartijas en cafetales y bosque primario en el Soconusco chiapaneco. *Rev. Mex. Biodivers.* **79**, 185–195.
102. MacSwiney MCG, Vilchis PL, Clarke FM, Racey PA. 2007 The importance of cenotes in conserving bat assemblages in the Yucatan, Mexico. *Biol. Conserv.* **136**, 499–509. (doi:10.1016/j.biocon.2006.12.021)
103. Mallari NAD, Collar NJ, Lee DC, McGowan PJK, Wilkinson R, Marsden SJ. 2011 Population densities of understorey birds across a habitat gradient in Palawan, Philippines: implications for conservation. *Oryx* **45**, 234–242. (doi:10.1017/s0030605310001031)
104. Malonza PK, Veith M. 2012 Amphibian community along elevational and habitat

disturbance gradients in the Taita Hills, Kenya. *Herpetotropicos* **7**, 7–16.
105. Marsh CJ, Lewis OT, Said I, Ewers RM. 2010 Community-level diversity modelling of birds and butterflies on Anjouan, Comoro Islands. *Biol. Conserv.* **143**, 1364–1374. (doi:10.1016/j.biocon.2010.03.010)
106. Martin PS, Gheler-Costa C, Lopes PC, Rosalino LM, Verdade LM. 2012 Terrestrial non-volant small mammals in agro-silvicultural landscapes of Southeastern Brazil. *For. Ecol. Manage.* **282**, 185–195. (doi:10.1016/j.foreco.2012.07.002)
107. McCarthy JL, McCarthy KP, Fuller TK, McCarthy TM. 2010 Assessing variation in wildlife biodiversity in the Tien Shan Mountains of Kyrgyzstan using ancillary camera-trap photos. *Mt. Res. Dev.* **30**, 295–301. (doi:10.1659/mrd-journal-d-09-00080.1)
108. McShea WJ, Stewart C, Peterson L, Erb P, Stuebing R, Giman B. 2009 The importance of secondary forest blocks for terrestrial mammals within an *Acacia*/secondary forest matrix in Sarawak, Malaysia. *Biol. Conserv.* **142**, 3108–3119. (doi:10.1016/j.biocon.2009.08.009)
109. Mena JL, Medellín R a. 2010 Small mammal assemblages in a disturbed tropical landscape at Pozuzo, Peru. *Mamm. Biol. – Zeitschrift für Säugetierkd.* **75**, 83–91. (doi:10.1016/j.mambio.2009.08.006)
110. Milder JC, DeClerck FAJ, Sanfiorenzo A, Sanchez DM, Tobar DE, Zuckerberg B. 2010 Effects of farm and landscape management on bird and butterfly conservation in western Honduras. *Ecosphere* **1**, art2. (doi:10.1890/es10-00003.1)
111. Miranda MV, Politi N, Rivera LO. 2010 Unexpected changes in the bird assemblage in areas under selective logging in piedmont forest in northwestern Argentina. *Ornitol. Neotrop.* **21**, 323–337.
112. Moreno-Mateos D, Rey Benayas JM, Perez-Camacho L, de la Montana E, Rebollo S, Cayuela L. 2011 Effects of land use on nocturnal birds in a Mediterranean agricultural landscape. *Acta Ornithol.* **46**, 173–182. (doi:10.3161/000164511x625946)
113. Munyekenye FB, Mwangi EM, Gichuki NN. 2008 Bird species richness and abundance in different forest types at Kakamega Forest, western Kenya. *Ostrich* **79**, 37–42. (doi:10.2989/ostrich.2008.79.1.4.361)
114. Naidoo R. 2004 Species richness and community composition of songbirds in a tropical forest-agricultural landscape. *Anim. Conserv.* **7**, 93–105.
115. Naithani A, Bhatt D. 2012 Bird community structure in natural and urbanized habitats along an altitudinal gradient in Pauri district (Garhwal Himalaya) of Uttarakhand state, India. *Biologia* **67**, 800–808. (doi:10.2478/s11756-012-0068-z)
116. Nakagawa M, Miguchi H, Nakashizuka T. 2006 The effects of various forest uses on small mammal communities in Sarawak, Malaysia. *For. Ecol. Manage.* **231**, 55–62. (doi:10.1016/j.foreco.2006.05.006)
117. Nakashima Y, Inoue E, Akomo-Okoue EF. 2013 Population density and habitat preferences of forest duikers in Moukalaba-Doudou National Park, Gabon. *African Zool.* **48**, 395–399. (doi:10.3377/004.048.0212)
118. Naoe S, Sakai S, Masaki T. 2012 Effect of forest shape on habitat selection of birds in a plantation-dominant landscape across seasons: comparison between continuous and strip forests. *J. For. Res.* **17**, 219–223. (doi:10.1007/s10310-011-0296-z)
119. Ndang'ang'a PK, Njoroge JBM, Githiru M. 2013 Vegetation composition and structure influences bird species community assemblages in the highland agricultural landscape of Nyandarua, Kenya. *Ostrich* **84**, 171–179. (doi:10.2989/00306525.2013.860929)
120. Neuschulz EL, Botzat A, Farwig N. 2011 Effects of forest modification on bird community composition and seed removal in a heterogeneous landscape in South

Africa. *Oikos* **120**, 1371–1379. (doi:10.1111/j.1600-0706.2011.19097.x)
121. Nicolas V, Barriere P, Tapiero A, Colyn M. 2009 Shrew species diversity and abundance in Ziama Biosphere Reserve, Guinea: comparison among primary forest, degraded forest and restoration plots. *Biodivers. Conserv.* **18**, 2043–2061. (doi:10.1007/s10531-008-9572-4)
122. O'Dea N, Whittaker RJ. 2007 How resilient are Andean montane forest bird communities to habitat degradation? *Biodivers. Conserv.* **16**, 1131–1159. (doi:10.1007/s10531-006-9095-9)
123. O'Farrell PJ, Donaldson JS, Hoffman MT, Mader AD. 2008 Small mammal diversity and density on the Bokkeveld escarpment, South Africa - implications for conservation and livestock predation. *African Zool.* **43**, 117–124. (doi: 10.3377/1562-7020(2008)43[117:SMDADO]2.0.CO;2)
124. Ofori-Boateng C, Oduro W, Hillers A, Norris K, Oppong SK, Adum GB, Rödel MO. 2013 Differences in the effects of selective logging on amphibian assemblages in three West African forest types. *Biotropica*. **45**, 94–101.
125. Otto CR V, Roloff GJ. 2012 Songbird response to green-tree retention prescriptions in clearcut forests. *For. Ecol. Manage.* **284**, 241–250. (doi:10.1016/j.foreco.2012.07.016)
126. Owiunji I, Plumptre a. . 1998 Bird communities in logged and unlogged compartments in Budongo Forest, Uganda. *For. Ecol. Manage.* **108**, 115–126. (doi:10.1016/S0378-1127(98)00219-9)
127. Paritsis J, Aizen MA. 2008 Effects of exotic conifer plantations on the biodiversity of understory plants, epigeal beetles and birds in Nothofagus dombeyi forests. *For. Ecol. Manage.* **255**, 1575–1583. (doi:10.1016/j.foreco.2007.11.015)
128. Parry L, Barlow JOS, Peres CA. 2009 Hunting for sustainability in tropical secondary forests. **23**, 1270–1280. (doi:10.1111/j.1523-1739.2009.01224.x)
129. Pearman PB. 2002 The scale of community structure: habitat variation and avian guilds in tropical forest understory. *Ecol. Monogr.* **72**, 19–39. (doi:10.2307/3100083)
130. Pelegrin N, Bucher EH. 2012 Effects of habitat degradation on the lizard assemblage in the Arid Chaco, central Argentina. *J. Arid Environ.* **79**, 13–19. (doi:10.1016/j.jaridenv.2011.11.004)
131. Peres CA, Nascimento HS. 2006 Impact of game hunting by the Kayapo of south-eastern Amazonia: implications for wildlife conservation in tropical forest indigenous reserves. *Biodivers. Conserv.* **15**, 2627–2653. (doi:10.1007/s10531-005-5406-9)
132. Pethiyagoda Rohan S. J, Manamendra-Arachchi K. 2012 Endangered anurans in a novel forest in the highlands of Sri Lanka. *Wildl. Res.* **39**, 641–648. (doi:10.1071/wr12079)
133. Phalan B, Onial M, Balmford A, Green RE. 2011 Reconciling food production and biodiversity conservation: land sharing and land sparing compared. *Science* **333**, 1289–1291. (doi:10.1126/science.1208742)
134. Pineda E, Halffter G. 2004 Species diversity and habitat fragmentation: frogs in a tropical montane landscape in Mexico. *Biol. Conserv.* **117**, 499–508. (doi:10.1016/j.biocon.2003.08.009)
135. Politi N, Hunter Jr. M, Rivera L. 2012 Assessing the effects of selective logging on birds in Neotropical piedmont and cloud montane forests. *Biodivers. Conserv.* **21**, 3131–3155. (doi:10.1007/s10531-012-0358-3)
136. Pons P, Wendenburg C. 2005 The impact of fire and forest conversion into savanna on the bird communities of West Madagascan dry forests. *Anim. Conserv.* **8**, 183–193. (doi:10.1017/s1367943005001940)
137. Presley SJ, Willig MR, Jr JMW, Saldanha LN. 2008 Effects of reduced-impact logging and forest physiognomy on bat populations of lowland Amazonian forest. *J. Appl.*

Ecol. **45**, 14–25. (doi:10.1111/j.1365-2664.2007.01373.x)
138. Proença VM, Pereira HM, Guilherme J, Vicente L. 2010 Plant and bird diversity in natural forests and in native and exotic plantations in NW Portugal. *Acta Oecologica* **36**, 219–226. (doi:10.1016/j.actao.2010.01.002)
139. Ranganathan J, Chan KMA, Daily GC. 2007 Satellite detection of bird communities in tropical countryside. *Ecol. Appl.* **17**, 1499–1510. (doi:10.1890/06-0285.1)
140. Ranganathan J, Daniels RJR, Chandran MDS, Ehrlich PR, Daily GC. 2008 Sustaining biodiversity in ancient tropical countryside. *Proc. Natl. Acad. Sci. U. S. A.* **105**, 17852–17854. (doi:10.1073/pnas.0808874105)
141. Reid JL, Harris JBC, Zahawi RA. 2012 Avian habitat preference in tropical forest restoration in Southern Costa Rica. *Biotropica* **44**, 350–359. (doi:10.1111/j.1744-7429.2011.00814.x)
142. Rey-Benayas JM, Galvan I, Carrascal LM. 2010 Differential effects of vegetation restoration in Mediterranean abandoned cropland by secondary succession and pine plantations on bird assemblages. *For. Ecol. Manage.* **260**, 87–95. (doi:10.1016/j.foreco.2010.04.004)
143. Reynolds C, Symes CT. 2013 Grassland bird response to vegetation structural heterogeneity and clearing of invasive bramble. *African Zool.* **48**, 228–239. (doi:10.3377/004.048.0217)
144. Richardson BA, Richardson MJ, Soto-Adames FN. 2005 Separating the effects of forest type and elevation on the diversity of litter invertebrate communities in a humid tropical forest in Puerto Rico. *J. Anim. Ecol.* **74**, 926–936. (doi:10.1111/j.1365-2656.2005.00990.x)
145. Rubio A V, Simonetti JA. 2011 Lizard assemblages in a fragmented landscape of central Chile. *Eur. J. Wildl. Res.* **57**, 195–199. (doi:10.1007/s10344-010-0434-5)
146. Saldaña-Vázquez RA, Sosa VJ, Hernández-Montero JR, López-Barrera F. 2010 Abundance responses of frugivorous bats (Stenodermatinae) to coffee cultivation and selective logging practices in mountainous central Veracruz, Mexico. *Biodivers. Conserv.* **19**, 2111–2124. (doi:10.1007/s10531-010-9829-6)
147. Sam K, Koane B, Jeppy S, Novotny V. 2014 Effect of forest fragmentation on bird species richness in Papua New Guinea. *J. F. Ornithol.* **85**, 152–167. (doi:10.1111/jofo.12057)
148. Santana J, Porto M, Gordinho L, Reino L, Beja P. 2012 Long-term responses of Mediterranean birds to forest fuel management. *J. Appl. Ecol.* **49**, 632–643. (doi:10.1111/j.1365-2664.2012.02141.x)
149. Scott DM, Brown D, Mahood S, Denton B, Silburn A, Rakotondraparany F. 2006 The impacts of forest clearance on lizard, small mammal and bird communities in the arid spiny forest, southern Madagascar. *Biol. Conserv.* **127**, 72–87. (doi:10.1016/j.biocon.2005.07.014)
150. Sedlock JL, Weyandt SE, Cororan L, Damerow M, Hwa S-H, Pauli B. 2008 Bat diversity in tropical forest and agro-pastoral habitats within a protected area in the Philippines. *Acta Chiropterologica* **10**, 349–358. (doi:10.3161/150811008x414926)
151. Shafie NJ, Sah SAM, Latip NSA, Azman NM, Khairuddin NL. 2011 Diversity pattern of bats at two contrasting habitat types along Kerian River, Perak, Malaysia. *Trop. Life Sci. Res.* **22**, 13–22.
152. Shahabuddin G, Kumar R. 2006 Influence of anthropogenic disturbance on birds of tropical dry forest: the role of vegetation structure. *Anim. Conserv.* **9**, 404–413. (doi:10.1111/j.1469-1795.2006.00051.x)
153. Sheldon FH, Styring A, Hosner PA. 2010 Bird species richness in a Bornean exotic tree plantation: A long-term perspective. *Biol. Conserv.* **143**, 399–407.

(doi:10.1016/j.biocon.2009.11.004)
154. Sodhi NS *et al.* 2010 Deforestation and avian extinction on tropical landbridge islands. *Conserv. Biol.* **24**, 1290–1298. (doi:10.1111/j.1523-1739.2010.01495.x)
155. Soh MCK, Sodhi NS, Lim SLH. 2006 High sensitivity of montane bird communities to habitat disturbance in Peninsular Malaysia. *Biol. Conserv.* **129**, 149–166. (doi:10.1016/j.biocon.2005.10.030)
156. Sosa RA, Benz VA, Galea JM, Poggio Herrero I V. 2010 Efecto del grado de disturbio sobre el ensamble de aves en la reserva provincial Parque Luro, La Pampa, Argentina. *Rev. la Asoc. Argentina Ecol. Paisajes* **1**, 101–110.
157. Sridhar H, Raman TRS, Mudappa D. 2008 Mammal persistence and abundance in tropical rainforest remnants in the southern Western Ghats, India. *Curr. Sci.* **94**, 748–757.
158. Stouffer PC, Johnson EI, Bierregaard Jr. RO, Lovejoy TE. 2011 Understory bird communities in Amazonian rainforest fragments: species turnover through 25 years post-isolation in recovering landscapes. *PLoS One* **6**. (doi:10.1371/journal.pone.0020543)
159. Struebig MJ, Kingston T, Zubaid A, Mohd-Adnan A, Rossiter SJ. 2008 Conservation value of forest fragments to Palaeotropical bats. *Biol. Conserv.* **141**, 2112–2126. (doi:10.1016/j.biocon.2008.06.009)
160. Suarez-Rubio M, Thomlinson JR. 2009 Landscape and patch-level factors influence bird communities in an urbanized tropical island. *Biol. Conserv.* **142**, 1311–1321. (doi:10.1016/j.biocon.2008.12.035)
161. Sung YH, Karraker NE, Hau BCH. 2012 Terrestrial herpetofaunal assemblages in secondary forests and exotic *Lophostemon confertus* plantations in South China. *For. Ecol. Manage.* **270**, 71–77. (doi:10.1016/j.foreco.2012.01.011)
162. Threlfall CG, Law B, Banks PB. 2012 Sensitivity of insectivorous bats to urbanization: implications for suburban conservation planning. *Biol. Conserv.* **146**, 41–52. (doi:10.1016/j.biocon.2011.11.026)
163. Urbina-Cardona JN, Olivares-Perez M, Reynoso VH. 2006 Herpetofauna diversity and microenvironment correlates across a pasture-edge-interior ecotone in tropical rainforest fragments in the Los Tuxtlas Biosphere Reserve of Veracruz, Mexico. *Biol. Conserv.* **132**, 61–75. (doi:10.1016/j.biocon.2006.03.014)
164. Urbina-Cardona JN, Londoño-Murcia MC, García-Ávila DG. 2008 Spatio-temporal dymanics of snake diversity in four habitats with different degrees of anthropogenic disturbance in the Gorgona Island National Natural Park in the Colombian Pacific. *Caldasia* **30**, 479–493.
165. Vallan D. 2002 Effects of anthropogenic environmental changes on amphibian diversity in the rain forests of eastern Madagascar. *J. Trop. Ecol.* **18**, 725–742. (doi:10.1017/S026646740200247X)
166. Vergara PM, Simonetti JA. 2004 Avian responses to fragmentation of the Maulino Forest in central Chile. *Oryx* **38**, 383–388. (doi:10.1017/s0030605304000742)
167. Verhulst J, Báldi A, Kleijn D. 2004 Relationship between land-use intensity and species richness and abundance of birds in Hungary. *Agric. Ecosyst. Environ.* **104**, 465–473. (doi:10.1016/j.agee.2004.01.043)
168. Waite E, Closs GP, van Heezik Y, Dickinson KJM. 2013 Resource availability and foraging of Silvereyes (*Zosterops lateralis*) in urban trees. *Emu* **113**, 26–32. (doi:10.1071/mu11093)
169. Walker S, Wilson DJ, Norbury G, Monks A, Tanentzap AJ. 2014 Complementarity of indigenous flora in shrublands and grasslands in a New Zealand dryland landscape. *N. Z. J. Ecol.* **38**, 230–241.

170. Wang Y, Bao Y, Yu M, Xu G, Ding P. 2010 Nestedness for different reasons: the distributions of birds, lizards and small mammals on islands of an inundated lake. *Divers. Distrib.* **16**, 862–873. (doi:10.1111/j.1472-4642.2010.00682.x)
171. Watling JI, Gerow K, Donnelly MA. 2009 Nested species subsets of amphibians and reptiles on Neotropical forest islands. *Anim. Conserv.* **12**, 467–476. (doi:10.1111/j.1469-1795.2009.00274.x)
172. Wells K, Kalko EK V., Lakim MB, Pfeiffer M. 2007 Effects of rain forest logging on species richness and assemblage composition of small mammals in Southeast Asia. *J. Biogeogr.* **34**, 1087–1099. (doi:10.1111/j.1365-2699.2006.01677.x)
173. Wiafe ED, Amfo-Otu R. 2012 Forest duiker (*Cephalophus* spp.) abundance and hunting activities in the Kakum conservation area, Ghana. *J. Ecol. Nat. Environ.* **4**, 114–118. (doi:10.5897/jene11.144)
174. Willig MR, Presley SJ, Bloch CP, Hice CL, Yanoviak SP, Díaz MM, Chauca LA, Pacheco V, Weaver SC. 2007 Phyllostomid bats of lowland Amazonia: effects of habitat alteration on abundance. *Biotropica* **39**, 737–746. (doi:10.1111/j.1744-7429.2007.00322.x)
175. Woinarski JCZ, Rankmore B, Hill B, Griffiths AD, Stewart A, Grace B. 2009 Fauna assemblages in regrowth vegetation in tropical open forests of the Northern Territory, Australia. *Wildl. Res.* **36**, 675–690. (doi:10.1071/wr08128)
176. Woinarski JCZ, Ash a. J. 2002 Responses of vertebrates to pastoralism, military land use and landscape position in an Australian tropical savanna. *Austral Ecol.* **27**, 311–323. (doi:10.1046/j.1442-9993.2002.01182.x)
177. Wunderle JM, Henriques LMP, Willig MR. 2006 Short-term responses of birds to forest gaps and understory: an assessment of reduced-impact logging in a lowland Amazon Forest. *Biotropica* **38**, 235–255.
178. Yamaura Y, Royle JA, Shimada N, Asanuma S, Sato T, Taki H, Makino S. 2012 Biodiversity of man-made open habitats in an underused country: a class of multispecies abundance models for count data. *Biodivers. Conserv.* **21**, 1365–1380. (doi:10.1007/s10531-012-0244-z)
179. Yoshikura S, Yasui S, Kamijo T. 2011 Comparative study of forest-dwelling bats' abundances and species richness between old-growth forests and conifer plantations in Nikko National Park, central Japan. *Mammal Study* **36**, 189–198. (doi:10.3106/041.036.0402)
180. Zimmerman G, Bell FW, Woodcock J, Palmer A, Paloniemi J. 2011 Response of breeding songbirds to vegetation management in conifer plantations established in boreal mixedwoods. *For. Chron.* **87**, 217–224.
181. Hillebrand H *et al.* 2018. Biodiversity change is uncoupled from species richness trends: consequences for conservation and monitoring. *J. Appl. Ecol.* (doi:10.1111/1365-2664.12959)
182. VanDerWal J, Shoo LP, Johnson CN, Williams SE. 2009 Abundance and the environmental niche: environmental suitability estimated from niche models predicts the upper limit of local abundance. *Am. Nat.* **174**, 282–291. (doi:10.1086/600087)
183. Hudson LN *et al.* 2014 The PREDICTS database: a global database of how local terrestrial biodiversity responds to human impacts. *Ecol. Evol.* **4**, 4701–4735. (doi:10.1002/ece3.1303)
184. Center for International Earth Science Information Network (CIESIN) Columbia University, International Food Policy Research Institute (IFPRI), The World Bank, Centro Internacional de Agricultura Tropical (CIAT). 2011 *Global Rural-Urban Mapping Project, Version 1 (GRUMPv1): Population Density Grid.* Palisades, NY, United States: NASA Socioeconomic Data and Applications Center (SEDAC). See:

http://sedac.ciesin.columbia.edu/data/dataset/grump-v1-population-density.
185. ESRI. 2015 ArcGIS Desktop: Version 10.3.
186. Bolker BM, Brooks ME, Clark CJ, Geange SW, Poulsen JR, Stevens MHH, White J-SS. 2008 Generalized linear mixed models: a practical guide for ecology and evolution. *Trends Ecol. Evol.* **24**, 127–135. (doi:10.1016/j.tree.2008.10.008)
187. Fournier DA, Skaug HJ, Ancheta J, Ianelli J, Magnusson A, Maunder MN, Nielsen A, Sibert J. 2012 AD Model Builder: using automatic differentiation for statistical inference of highly parameterized complex nonlinear models. *Optim. Methods Softw.* **27**, 233–249.
188. van Vuuren DP *et al.* 2011 The representative concentration pathways: an overview. *Clim. Change* **109**, 5–31. (doi:10.1007/s10584-011-0148-z)
189. Burrows MT *et al.* 2014 Geographical limits to species-range shifts are suggested by climate velocity. *Nature* **507**, 492–495. (doi:10.1038/nature12976)
190. Oldfield F, Steffen W. 2014 Anthropogenic climate change and the nature of Earth System science. *Anthr. Rev.* **1**, 70–75. (doi:10.1177/2053019613514862)
191. Harfoot M, Tittensor DP, Newbold T, McInerny G, Smith MJ, Scharlemann JPW. 2014 Integrated assessment models for ecologists: the present and the future. *Glob. Ecol. Biogeogr.* **23**, 124–143. (doi:10.1111/geb.12100)
192. Hurtt GC *et al.* 2011 Harmonization of land-use scenarios for the period 1500–2100: 600 years of global gridded annual land-use transitions, wood harvest, and resulting secondary lands. *Clim. Change* **109**, 117–161. (doi:10.1007/s10584-011-0153-2)
193. Asselen S, Verburg PH. 2012 A Land System representation for global assessments and land-use modeling. *Glob. Chang. Biol.* **18**, 3125–3148. (doi:10.1111/j.1365-2486.2012.02759.x)
194. Liu C, Berry PM, Dawson TP, Pearson RG. 2005 Selecting thresholds of occurrence in the prediction of species distributions. *Ecography* **3**, 385–393.
195. Svenning J-C, Skov F. 2004 Limited filling of the potential range in European tree species. *Ecol. Lett.* **7**, 565–573. (doi:10.1111/j.1461-0248.2004.00614.x)
196. Gonzalez P. 2001 Desertification and a shift of forest species in the West African Sahel. *Clim. Res.* **17**, 217–228. (doi:10.3354/cr017217)
197. Whitmee S, Orme CDL. 2013 Predicting dispersal distance in mammals: a trait-based approach. *J. Anim. Ecol.* **82**, 211–221. (doi:10.1111/j.1365-2656.2012.02030.x)
198. Sutherland GD, Harestad AS, Price K, Lertzman KP. 2000 Scaling of natal dispersal distances in terrestrial birds and mammals. *Conserv. Ecol.* **4**, 16.
199. Paradis E, Baillie SR, Sutherland WJ, Gregory RD. 1998 Patterns of natal and breeding dispersal in birds. *J. Anim. Ecol.* **67**, 518–536. (doi:10.1046/j.1365-2656.1998.00215.x)
200. Frishkoff LO, Hadly EA, Daily GC. 2015 Thermal niche predicts tolerance to habitat conversion in tropical amphibians and reptiles. *Glob. Chang. Biol.* **21**, 3901–3916. (doi:10.1111/gcb.13016)
201. Frishkoff LO, Karp DS, Flanders JR, Zook J, Hadly EA, Daily GC, M'Gonigle LK. 2016 Climate change and habitat conversion favour the same species. *Ecol. Lett.* **19**, 1081–1090. (doi:10.1111/ele.12645)
202. Pearson RG, Dawson TP. 2003 Predicting the impacts of climate change on the distribution of species: are bioclimate envelope models useful? *Glob. Ecol. Biogeogr.* **12**, 361–371. (doi:10.1046/j.1466-822X.2003.00042.x)
203. Olson DM *et al.* 2001 Terrestrial ecoregions of the world: a new map of life on Earth. *Bioscience* **51**, 933–938. (doi:10.1641/0006-3568(2001)051[0933:TEOTWA]2.0.CO;2)
204. Rogelj J, Meinshausen M, Knutti R. 2012 Global warming under old and new

scenarios using IPCC climate sensitivity range estimates. *Nat. Clim. Chang.* **2**, 248–253. (doi:10.1038/nclimate1385)
